# CRISPR/Cas9 gene editing for the creation of an MGAT1-deficient CHO cell line to control HIV-1 vaccine glycosylation

**DOI:** 10.1371/journal.pbio.2005817

**Published:** 2018-08-29

**Authors:** Gabriel Byrne, Sara M. O’Rourke, David L. Alexander, Bin Yu, Rachel C. Doran, Meredith Wright, Qiushi Chen, Parastoo Azadi, Phillip W. Berman

**Affiliations:** 1 Department of Biomolecular Engineering, University of California Santa Cruz, Santa Cruz, California, United States of America; 2 Complex Carbohydrate Research Center, University of Georgia, Athens, Georgia, United States of America; Weatherall Institute of Molecular Medicine, University of Oxford, United States of America

## Abstract

Over the last decade, multiple broadly neutralizing monoclonal antibodies (bN-mAbs) to the HIV-1 envelope protein (Env) gp120 have been described. Many of these recognize epitopes consisting of both amino acid and glycan residues. Moreover, the glycans required for binding of these bN-mAbs are early intermediates in the N-linked glycosylation pathway. This type of glycosylation substantially alters the mass and net charge of Envs compared to molecules with the same amino acid sequence but possessing mature, complex (sialic acid–containing) carbohydrates. Since cell lines suitable for biopharmaceutical production that limit N-linked glycosylation to mannose-5 (Man_5_) or earlier intermediates are not readily available, the production of vaccine immunogens displaying these glycan-dependent epitopes has been challenging. Here, we report the development of a stable suspension-adapted Chinese hamster ovary (CHO) cell line that limits glycosylation to Man_5_ and earlier intermediates. This cell line was created using the clustered regularly interspaced short palindromic repeat (CRISPR)/CRISPR-associated protein 9 (Cas9) gene editing system and contains a mutation that inactivates the gene encoding Mannosyl (Alpha-1,3-)-Glycoprotein Beta-1,2-N-Acetylglucosaminyltransferase (MGAT1). Monomeric gp120s produced in the MGAT1^−^ CHO cell line exhibit improved binding to prototypic glycan-dependent bN-mAbs directed to the V1/V2 domain (e.g., PG9) and the V3 stem (e.g., PGT128 and 10–1074) while preserving the structure of the important glycan-independent epitopes (e.g., VRC01). The ability of the MGAT1^−^ CHO cell line to limit glycosylation to early intermediates in the N-linked glycosylation pathway without impairing the doubling time or ability to grow at high cell densities suggests that it will be a useful substrate for the biopharmaceutical production of HIV-1 vaccine immunogens.

## Author summary

Though there is no HIV-1 vaccine available yet, significant progress has been made in understanding the envelope protein structure and the antibodies that bind to it. While most secreted or cell surface eukaryotic proteins contain several large, complex sugar groups, the HIV-1 envelope protein is covered in dense groups of polysaccharides. These sugars are of an intermediate, “high-mannose” form not typically found on eukaryotic proteins. A number of potent antibodies against HIV-1 have been discovered that specifically require these intermediate sugars to bind. This presents a challenge for vaccine production, as the cells used to produce most biopharmaceutical proteins, including previous HIV-1 vaccine candidates, have been selected to incorporate fully processed sugar groups, beyond the intermediate form found on the envelope protein. To address this problem, we used the clustered regularly interspaced palindromic repeat (CRISPR)/CRISPR-associated protein 9 (Cas9) gene editing system to create a Chinese hamster ovary (CHO) cell line that limits the sugar processing to the intermediate, high-mannose form. This paper describes the gene editing process, cell line selection, and antibody binding to the HIV-1 envelope produced. This line is capable of producing envelope proteins that bind the sugar-dependent antibodies, while possessing acceptable growth and production volume characteristics for large-scale manufacturing.

## Introduction

Despite 30 years of research, a vaccine capable of providing protection against human immunodeficiency virus type 1 (HIV-1) has yet to be described. However, considerable progress toward this goal has been achieved with the elucidation of the 3-dimensional structure of the HIV-1 envelope proteins (Envs; monomeric gp120 and trimeric gp140) and the characterization of multiple broadly neutralizing monoclonal antibodies (bN-mAbs) [1–5]. As headway toward a protective vaccine continues, the practicalities of large-scale vaccine production must be addressed. A growing body of evidence indicates that the N-linked glycosylation structure will be a critical factor in both the design and manufacture of any HIV vaccine [6–8].

Beginning in 2009, we learned that multiple bN-mAbs recognized glycan-dependent epitopes on the Env gp120. In an unanticipated development, several families of bN-mAbs require mannose-5 (Man_5_) and/or mannose-9 (Man_9_) for binding to key epitopes of gp120 [6,9–11]. As these bN-mAbs were being described, the data from the RV144 HIV vaccine trial were released. This study provided evidence for the first time that vaccination could prevent HIV infection in humans [12]. The regimen used in this trial involved immunization with a bivalent gp120 vaccine (AIDSVAX B/E) to stimulate an antibody response as well as immunization with a recombinant canarypox vector to stimulate a cell-mediated immune response [13–15]. This immunization protocol resulted in modest (31.2%) but significant vaccine efficacy [12]. Examination of the gp120 subunit vaccines used in the RV144 trial showed that both components (MN-recombinant gp120 [rgp120] and A244-rgp120) were enriched for complex, sialic acid–containing glycans and lacked the high-mannose glycosylation found on the surface of virions and native envelope proteins required to bind the new class of glycan-dependent bN-mAbs [16–20]. Thus, differences in glycosylation between the vaccine immunogens from the RV144 trial and virus particles could, in part, explain the low efficacy of RV144 and other gp120-based vaccines and their inability to elicit bN-mAbs. Previously, we reported that the same gp120s used in the RV144 trial could be modified to bind multiple bN-mAbs when expressed in a cell line (human embryonic kidney 293 [HEK 293] N-acetylglucosaminyltransferase I [GnTI]^−^) that limited N-linked glycosylation to Man_5_ or earlier species (e.g., Man_8_, Man_9_) [21]. While in theory this cell line could be used to produce a glycan-optimized gp120 vaccine, in reality, this is not practical. The HEK 293 GnTI^−^ system is not suitable for clinical and large-scale production, because of genetic instability and the inability to grow for sustained periods at high cell densities [22,23].

Chinese hamster ovary (CHO) cells have long been the substrate of choice for the production of therapeutic glycoproteins. This is due to their ability to grow at high densities in serum-free suspension cultures, sustain high levels of protein expression over prolonged fermentation cycles, and incorporate complex glycans on exogenously expressed proteins [24–26]. Typical glycoproteins contain only a few N-linked glycans, which aid in protein folding and intracellular trafficking. When these glycans terminate in sialic acid residues, they increase resistance to proteolysis and extend serum half-life in vivo [27–29]. Because of these physical and pharmacokinetic benefits, recombinant glycoprotein expression efforts have historically focused on maximizing the amount of complex, sialic acid–containing glycans per molecule. Although modern production technology provides the means to express and purify properly folded recombinant glycoproteins at a large scale, controlling the glycosylation has been a persistent problem for most glycoproteins because of the “non-templated” nature of glycosylation [30–32]. The final glycan structure of proteins with only a few N-linked glycosylation sites can be highly variable with respect to the glycan structure branching, saccharides present, sialic acid content, and net charge. Glycosylation heterogeneity is known to result from a variety of variables, including cell type, protein expression levels, cell culture conditions, monosaccharide donor availability, and protein structure [30,33–36]. Controlling glycosylation heterogeneity in gp120 is particularly problematic due to the fact that it contains an average of 25 potential N-linked glycosylation sites (PNGSs), constituting approximately 50% of the mass of the mature protein [37–40]. Each glycan site may be different in composition than others on the same molecule or different at the same position from molecule to molecule. Variance is so great that 79 different glycan structures have been found to occur at a single position in envelope proteins expressed in normal CHO cells [41].

In this paper, we address the problems of glycosylation heterogeneity and bN-mAb binding in the large-scale production of recombinant envelope proteins by the development of a mutant CHO cell line (MGAT1^−^ CHO) in which the Mannosyl (Alpha-1,3-)-Glycoprotein Beta-1,2-N-Acetylglucosaminyltransferase (MGAT1) gene has been inactivated using clustered regularly interspaced short palindromic repeat (CRISPR)/CRISPR-associated protein 9 (Cas9) gene editing. The nomenclature for the MGAT1 gene has changed over the years and was previously referred to as the GnTI gene. Inactivation or deficiency of the MGAT1 limits N-linked glycosylation to early oligomannose glycans (Man_5-9_) and enhances the binding of bN-mAbs to glycan-dependent epitopes, as compared to earlier gp120 vaccines produced in normal CHO cells. Although other CHO cell lines, such as CHO Lec1, that similarly limit oligomannose structures have been described, they grow slowly and differ from parental cell lines in morphology and growth characteristics [42,43]. Thus, the development of a precision-engineered CHO cell line resulting from CRISPR/Cas9 gene editing is a desirable alternative for HIV vaccine manufacturing. This cell line should be useful for the production of stable cell lines suitable for the production of HIV vaccines as well as other biopharmaceuticals for which limiting the incorporation of sialic acid is beneficial.

## Results

### Silencing of the CHO-S MGAT1 gene

**Fig 1:**
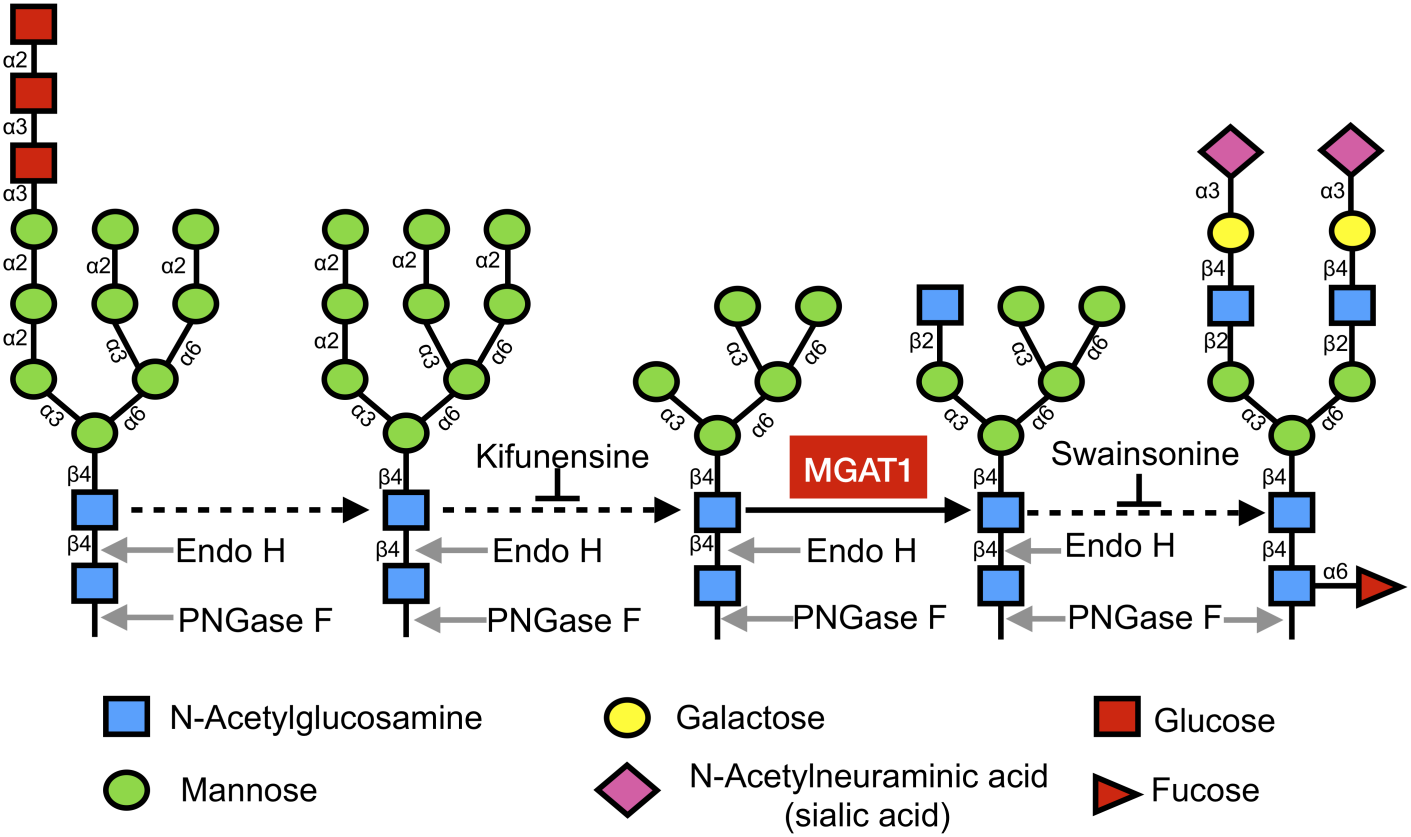
Simplified view of N-linked glycosylation pathway. N-linked glycosylation begins in the ER with the en-block transfer of a highly conserved Glc_3_Man_9_GlcNAc_2_ structure (left) to asparagine residues within the N-X-S/T motif of nascent proteins. This initial structure is sequentially trimmed to Man_9_GlcNAc_2_ and then Man_5_GlcNAc_2_ (center) as the protein moves from the ER to the Golgi apparatus. The enzyme MGAT1 (red box) adds a GlcNAc to the Man_5_ structure and is required to enable other glycosyltransferases to add monosaccharides, creating hybrid (second from right) and complex (right) glycoforms. Treatment with Endo H cleaves simple, oligomannose-containing glycans from glycoproteins, but not complex, sialic acid–containing glycans. PNGase F removes both simple and complex glycans from glycoproteins (indicated by the arrows). Kifunensine and swainsonine are inhibitors that halt processing at the steps indicated. Dashed black arrows indicate multiple enzymatic steps [47]. Endo H, endoglycosidase H; ER, endoplasmic reticulum; GlcNAc, N-acetylglucosamine; Man_5_, mannose-5_;_ Man_9_, mannose-9_;_ MGAT1, Mannosyl (Alpha-1,3-)-Glycoprotein Beta-1,2-N-Acetylglucosaminyltransferase; N-X-S/T, asparagine-X-serine/threonine; PNGase F, Peptide:N-Glycosidase F.

**Fig 2:**
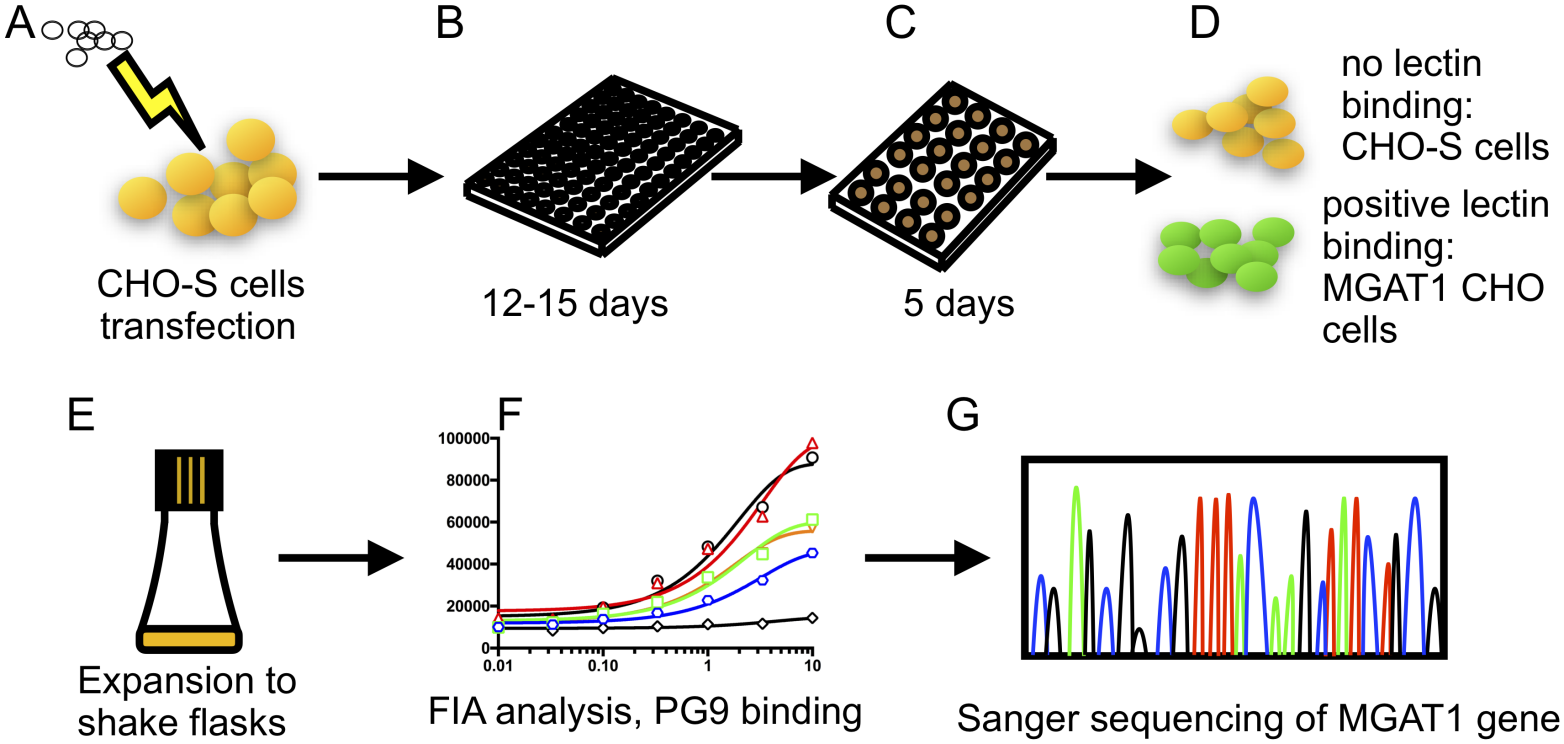
Flow chart of MGAT1 gene editing and cell line selection strategy. (A) A plasmid containing the Cas9 nuclease, tracrRNA, and a gRNA sequence was electroporated into suspension adapted CHO-S cells. (B) Twenty-four hours following transfection, the cells were distributed into 96-well tissue culture plates at a density of 0.5 cells/well. (C) Between 12 and 15 days later, wells with 20% or greater confluency were transferred to 24-well plates. (D) After 5 days of growth in 24-well plates, a 0.2-mL aliquot was removed from each well, and cells were tested for the ability to bind fluorescein-labeled GNA. (E) GNA-binding cells were then expanded to shake flasks, and cell lines were transiently transfected with a gene encoding A244-rgp120. The cell culture supernatants were then collected after 5 days and tested for binding of gp120 to the prototypic glycan-dependent, broadly neutralizing monoclonal antibody PG9. This representative plot (F) is shown for demonstrative process purposes only. A detailed plot of this data is show in Fig 4A. (G) The gene encoding MGAT1 was sequenced from GNA-binding cell lines that exhibited robust growth and the ability to secrete PG9-binding gp120. The specific mutations induced by NHEJR were determined by Sanger sequencing. Cas9, CRISPR-associated protein 9; CHO, Chinese hamster ovary; FIA, fluorescence immunoassay; GNA, *Galanthus nivalis* lectin; gRNA, guide RNA; MGAT1, Mannosyl (Alpha-1,3-)-Glycoprotein Beta-1,2-N-Acetylglucosaminyltransferase; NHEJR, nonhomologous end joining repair; rgp120, recombinant gp120; tracrRNA, *trans*-activating CRISPR RNA.

The goal of this project was to make an MGAT1-deficient CHO-S cell line using CRIPSR/Cas9. With this gene knocked out, complex, sialic acid–containing glycans cannot be formed, and N-linked glycosylation is not processed beyond the oligomannose Man_5_ structure (Fig 1). The CRISPR/Cas9 gene editing system allows for specific targeting of genes for deletion or modification by introducing double-stranded breaks (DSBs) followed by nonhomologous end joining (NHEJ) or homology-directed repair (HDR) [44,45]. We utilized a CRISPR/Cas9 nuclease vector containing an orange fluorescent protein (OFP) reporter gene (Materials and methods). After insertion of guide sequences, the vector contained all of the elements needed to induce a DSB in the MGAT1 gene. The sequence of the CHO MGAT1 gene was identified from GenBank, gene ID: 100682529 [46]. Three target-specific double-stranded guide sequences were ligated into the vector between a U6 promoter and a *trans*-activating CRISPR RNA (tracrRNA) sequence. The same vector encodes the Cas9 endonuclease and an OFP reporter gene, separated by a self-cleaving 2A peptide linker. This system allows for a single plasmid to encode for both the Cas9 and a complete guide RNA (gRNA), enabling the use of non-Cas9-expressing cells. Following ligation of these guide sequences, the vectors were transfected into CHO-S cells using the MaxCyte electroporation system (Fig 2). Targets 1 and 2 were introduced individually; the target 3 plasmid was mixed and added together in an equal ratio with target 2, creating 3 separate pools of transfected cells. Twenty-four hours post transfection, samples were serially diluted across five 96-well flat-bottom plates at a calculated density of 0.5 cells per well. The plates were examined daily, and wells with more than a single colony were discarded. Across the 15 total plates, between 15 and 30 wells per plate contained single viable colonies that were transferred to 24-well plates upon reaching 20% confluency after 12 to 15 days. Wells in the 96-well plates that did not have at least several dozen cells by day 15 were discarded. A total of 166 colonies were expanded to 24-well plates: 55 from the target 1 pool, 67 from the target 2 pool, and 44 from the combined target 2 and 3 pools.

### Lectin binding to detect MGAT1 gene inactivation

**Fig 3:**
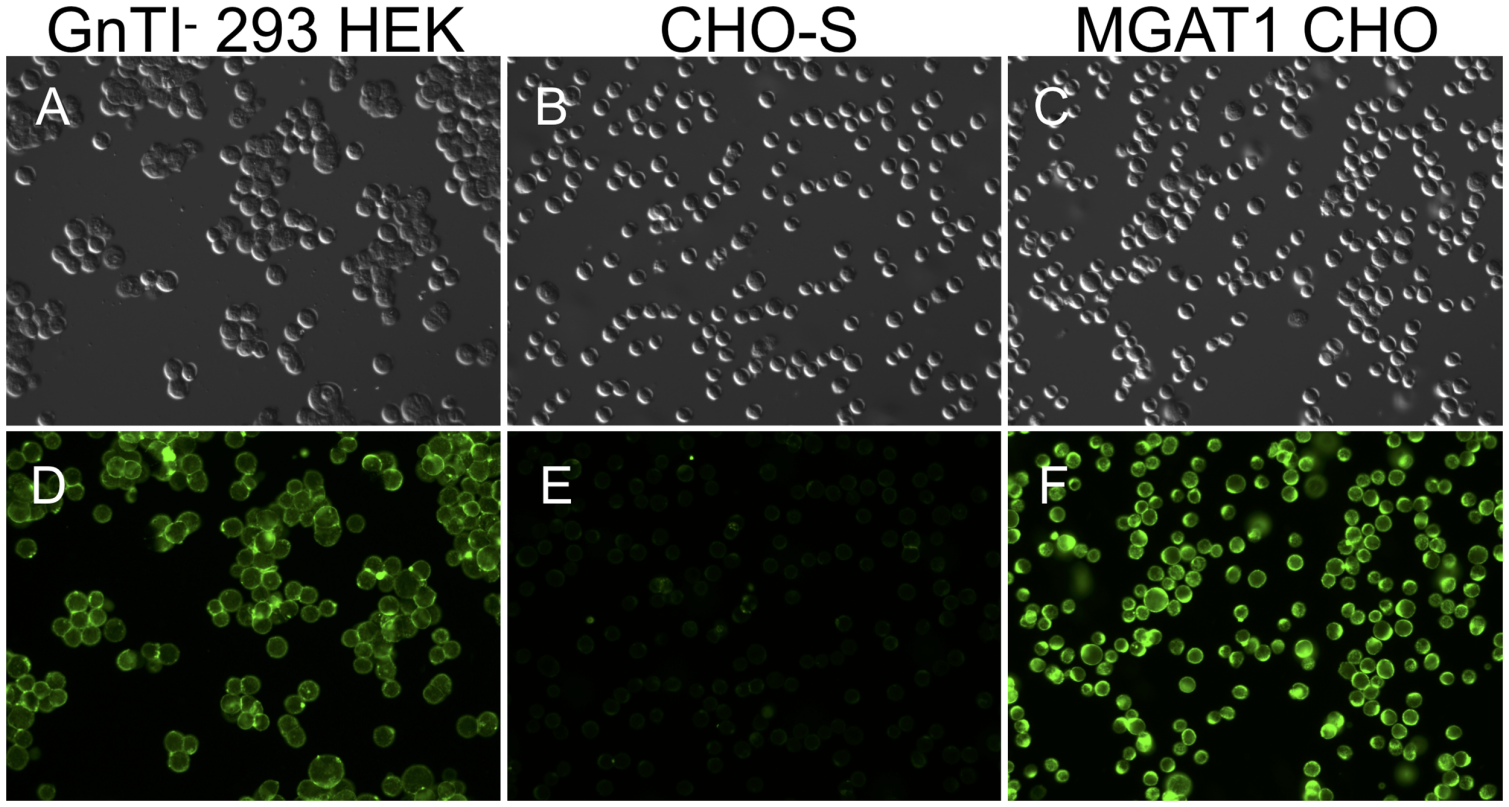
GNA probe for cell surface oligomannose glycan expression. GNA binds glycan structures with terminal mannose and will not bind complex, sialic acid–containing glycans. CHO-S cells were transfected with a plasmid designed to inactivate the MGAT1 gene by CRISPR/Cas9 gene editing (MGAT CHO). The cells were treated with fluorescein-conjugated GNA to screen for the incorporation of high-mannose glycans in the cell membrane. HEK 293 GnTI^−^ cells that also lack the MGAT1 gene served as a positive control (panels A and D), while normal CHO-S cells that possess an intact MGAT1 gene served as a negative control (panels B and E). Cells were visualized under 20× magnification on a Leica DM5500 B widefield microscope using DIC (upper panels A, B, C) or under illumination with 495-nm light (lower panels D, E, and F). Cas9, CRISPR-associated protein 9; CHO, Chinese hamster ovary; CRISPR, clustered regularly interspaced short palindromic repeat; DIC, differential interference contrast; GNA, *Galanthus nivalis* lectin; GnTI, N-acetylglucosaminyltransferase I; HEK 293, human embryonic kidney 293; MGAT1, Mannosyl (Alpha-1,3-)-Glycoprotein Beta-1,2-N-Acetylglucosaminyltransferase.

If the MGAT1 gene is inactivated, we expect glycoproteins to possess exclusively oligomannose forms of N-linked glycosylation, with a preponderance of Man_5_ isoforms on cell surface and secreted proteins. *Galanthus nivalis* lectin (GNA) recognizes glycans with terminal alpha-D mannose and is unable to bind to sialic acid–containing, complex glycans [48]. Accordingly, we used fluorescein-conjugated GNA to determine whether CRISPR/Cas9-transfected cells possessed a phenotype characteristic of cells with an inactivated MGAT1 gene. GNA does not require Ca^2+^ or Mg^2+^ cofactors to bind, allowing the use of 10 μM EDTA to ameliorate cell clumping during repeated centrifugation and wash steps. While MGAT1^−^ CHO cells and control HEK 293 GnTI^−^ cells bound to the GNA, the wild-type CHO-S cell line did not (Fig 3). A total of 20 GNA-binding cell lines from the original 166 candidates were selected on the basis of uniform GNA binding, and the cultures were expanded for further analysis. Three days following initial GNA selection, the 20 cell line candidates were reexamined, and 6 were rejected for lack of uniform lectin binding across the sample population, leaving 14 candidates.

### Expression of gp120 in MGAT1^−^ CHO cell lines

**Fig 4:**
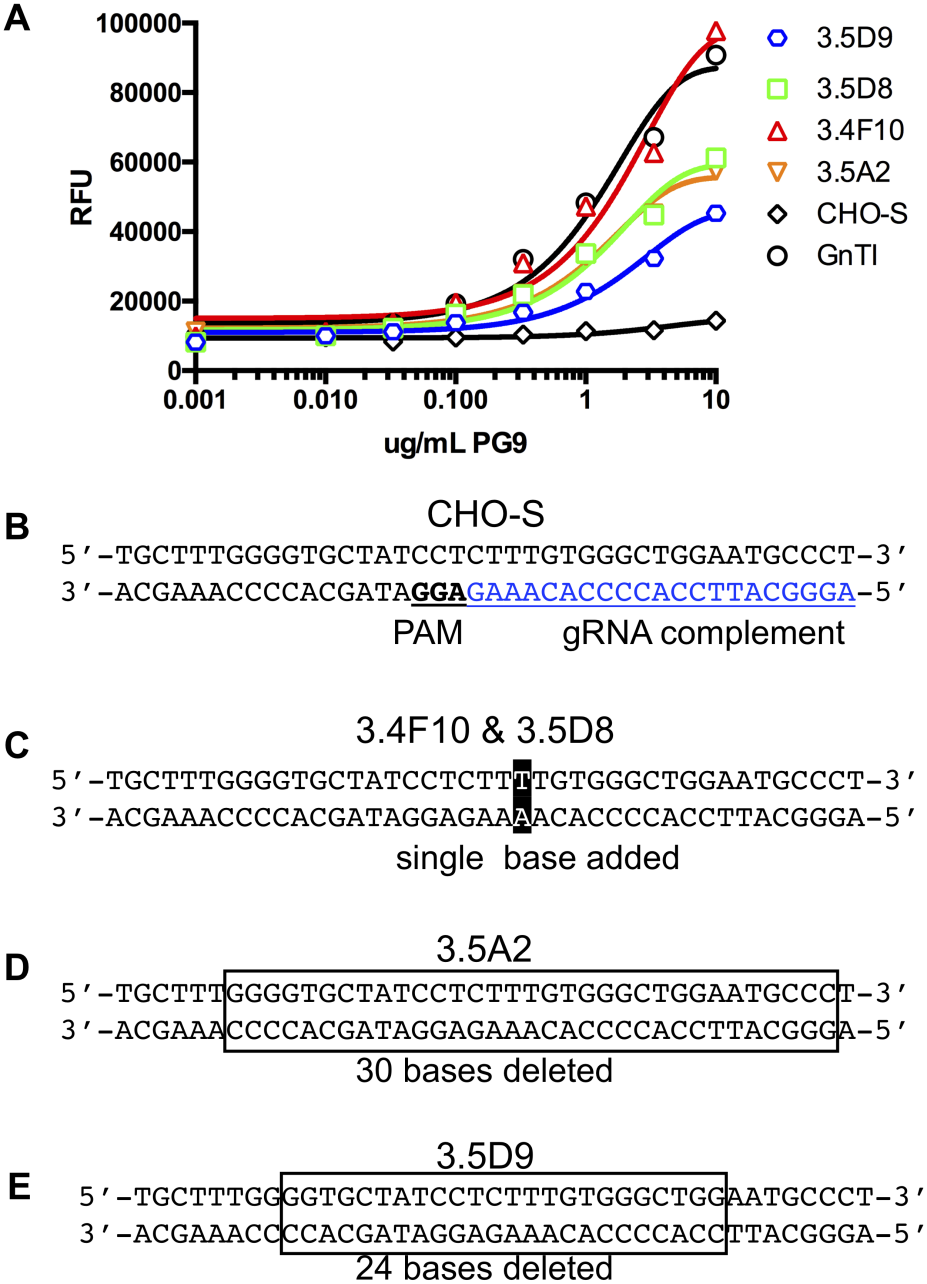
Screening and sequence analysis of the MGAT1^−^ CHO cell line. Colonies selected after MGAT1 gene inactivation were transiently transfected with a gene encoding A244-rgp120. Cell culture supernatants were collected and tested for binding by the glycan-dependent bN-mAb PG9. Based on PG9 binding studies, the MGAT genes from selected cell lines were amplified by PCR and sequenced. (A) PG9 binding to gp120 in cell culture supernatants of transiently transfected MGAT1^−^ CHO lines 3.5D9, 3.5D8, 3.4F10, and 3.5A2 and by supernatants from gp120-transfected CHO-S and HEK 293 GnTI^−^ cells. Underlaying data can be found in S1 Data. (B) Diagram of the unaltered CHO-S MGAT1 gene target section with gRNA complement sequence shown in blue and the PAM underlined in bold type. (C) Sequences of the MGAT1 gene for 3.4F10 and 3.5D8 cell lines both had the same single-base insertion, shown in a black box. (D) The sequence from the cell line 3.5A2 with bases deleted shown in a box. (E) The bases deleted from the 3.5D9 cell line sequences are indicated by the box. bN-mAb, broadly neutralizing monoclonal antibody; CHO, Chinese hamster ovary; GNTI, N-acetylglucosaminyltransferase I; gRNA, guide RNA; GnTI, N-acetylglucosaminyltransferase I; HEK 293, human embryonic kidney 293; MGAT1, Mannosyl (Alpha-1,3-)-Glycoprotein Beta-1,2-N-Acetylglucosaminyltransferase; PAM, protospacer-adjacent motif; RFU, relative fluorescence unit; rgp120, recombinant gp120.

Based on positive lectin binding criteria, the 14 candidate cell lines were grown in 125-mL shake flasks (Fig 2). Of those, the 4 fastest growing (3.4F10, 3.5D8, 3.5A2, and 3.4D9) were utilized for transient transfection with a gene encoding gp120 from the A244 strain of HIV-1 (A244-rgp120). Also transfected was the CHO-S parental cell line for comparison. This gp120 A244 had the sequence point mutations E332N and N334S, introducing a PNGS at N332. Five days post transfection, the culture media were harvested, and secreted gp120 proteins were purified by immunoaffinity chromatography. The purified products were assayed for protein yield and ability to bind the glycan-dependent broadly neutralizing antibody (bNAb) PG9 by fluorescent immune assay (FIA) (Fig 4). Previous studies have shown that this bNAb requires Man_5_ at position N160 in the V1/V2 domain for binding [3]. The results from this study confirmed that the MGAT1^−^ CHO cell lines could bind this antibody, whereas gp120 produced in the parental CHO-S cell line was unable to bind PG9. From this analysis, a single MGAT1^−^ CHO cell line, 3.4F10, was selected for further characterization and analysis (Fig 4).

To be a viable substrate for biopharmaceutical production, the growth and protein yield of the knockout line had to be comparable to the parental line. In transient transfection experiments, the calculated recovery of purified protein was 35.4 mg/L for the 3.4F10 MGAT1^−^ CHO line and 32.2 mg/L for the parental CHO-S line. Production of the same protein in HEK 293 GnTI^−^ cells by transient transfection yielded 1.9 mg/L. We measured that the cell-doubling time for the 3.4F10 MGAT1^−^ CHO cell line in BalanCD CHO Growth A media was 20.7 hours in a 1-L shaker flask during logarithmic growth phase, reaching a density of 1.9 × 10^7^ cells/mL. This was similar to the parental CHO-S population doubling time of 19.0 hours that achieved cell densities of 1.6 × 10^7^ cells/mL. By comparison, the HEK 293 GnTI^−^ cell line had a logarithmic cell-doubling time of 23.3 hours and achieved a maximal cell density of 4.20 × 10^6^ cells/mL when grown in Freestyle 293 media.

### Identification of CRISPR/Cas9-induced genetic alteration

To confirm that the MGAT1 gene had been inactivated, we sequenced the gene from the 3.4F10 line and the next 3 best candidates. An extra thymidine had been inserted at the Cas9 cleavage site of the 3.4F10 line MGAT1 gene, introducing a frameshift mutation. This mutation resulted in 23 altered codons and the insertion of a premature stop codon. The 3.5D8 line contained the same mutation, while 3.5D9 and 3.5A2 both had in-frame deletions of 24 and 30 nucleotides, respectively. The deleted codons of 3.5D9 and 3.5A2 corresponded to the transmembrane domain of the GnTI protein, leaving the active extracellular domain intact. The diminished binding of gp120s produced in the 3.5D9 and 3.5 A2 clones to PG9 suggest that partial MGAT1 activity remains in these 2 clones, compared to the 3.4F10 clone that, like gp120 produced in GnTI^−^ cells, exhibits improved binding to PG9 (Fig 4). Given that the single base insertion in the 3.4F10 MGAT1 gene resulted in a frameshift 51 nucleotides into a 1,276-bp-long gene, it is highly unlikely that the function of this gene could be restored by random mutation.

### Characterization of MGAT1^−^ CHO gp120 glycosylation

**Fig 5:**
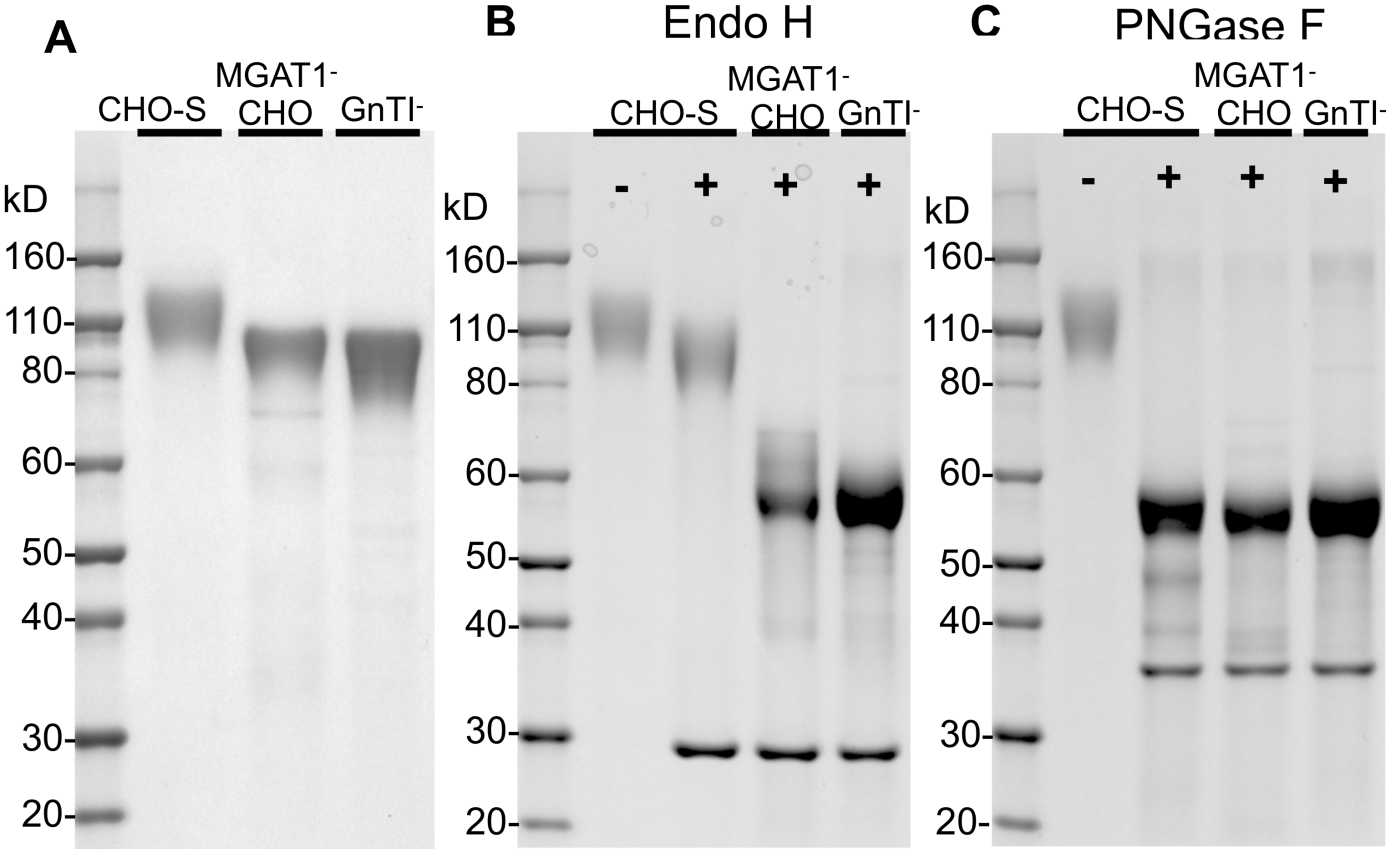
Endoglycosidase analysis of gp120 produced in MGAT1^−^ CHO cell line. Purified A244-rgp120 recovered from transiently transfected CHO-S, MGAT1^−^ CHO, or HEK 293 GnTI^−^ cell lines was analyzed by SDS-PAGE following endoglycosidase treatment. Purified gp120s were reduced, denatured, and then treated with either Endo H or PNGase F. The digests were then analyzed on 4%–12% tris-glycine SDS-PAGE gels and stained with Coomassie blue dye. Panel A, mock digests of gp120s produced in CHO-S, MGAT1^−^ CHO, and HEK 293 GnTI^−^ cells. Panel B, the same proteins in panel A, digested with Endo H. Panel C, the same proteins in panel A, digested with PNGase F. The mobility of molecular weight markers is shown for each gel. The endoglycosidase proteins are visible as bands at 29 kD (Endo H, panel B) and 36 kD (PNGase F, panel C). CHO, Chinese hamster ovary; Endo H, endoglycosidase H; GnTI, N-acetylglucosaminyltransferase I; HEK 293, human embryonic kidney 293; MGAT1, Mannosyl (Alpha-1,3-)-Glycoprotein Beta-1,2-N-Acetylglucosaminyltransferase; PNGase F, Peptide:N-Glycosidase F; rgp120, recombinant gp120.

Two additional methods (endoglycosidase digestion and mass spectrometry analysis using matrix-assisted laser desorption ionization–time of flight mass spectrometry [MALDI-TOF-MS]) were used to further characterize the N-linked glycosylation incorporated in A244-rgp120 produced by the 3.4F10 MGAT1^−^ CHO cell line. Immunoaffinity-purified, monomeric A244 gp120s produced by the CHO-S, HEK 293 GnTI^−^, and MGAT1^-^ CHO cell lines were digested overnight by Peptide:N-Glycosidase F (PNGase F) and endoglycosidase H (Endo H) and then analyzed by SDS-PAGE and stained with Coomassie blue dye (Fig 5). Endo H cleaves N-linked high-mannose glycan structures and not complex, sialic acid–containing glycans. When the protein produced in the HEK 293 GnTI^−^ and MGAT1^−^ CHO cell lines was compared to the proteins produced in CHO-S cell lines, we noted a reduction in mass of approximately 20 kD. This is in keeping with the smaller mass of the Man_5_ glycoform compared to that of the hybrid and complex glycans found on CHO-S-produced material. Following Endo H digestion, the protein produced in the CHO-S cell line was largely unaltered, indicating that it possessed the normal complex, sialic acid–containing glycans. In contrast, the proteins produced in the MGAT1^−^ CHO and HEK 293 GnTI^−^ cells were reduced to approximately 60 kD in size. This result was consistent with the observation that approximately half the mass of a given gp120 molecule can be attributed to N-linked glycosylation [47,49,50]. The complete sensitivity of the proteins produced in the MGAT1^−^ CHO and HEK 293 GnTI^−^ cells to Endo H digestion suggests that the glycosylation of these cell lines is exclusively high mannose. When digested with PNGase F, all samples dropped to the same size, confirming that undigested gp120 size variances were due to glycosylation differences.

**Fig 6:**
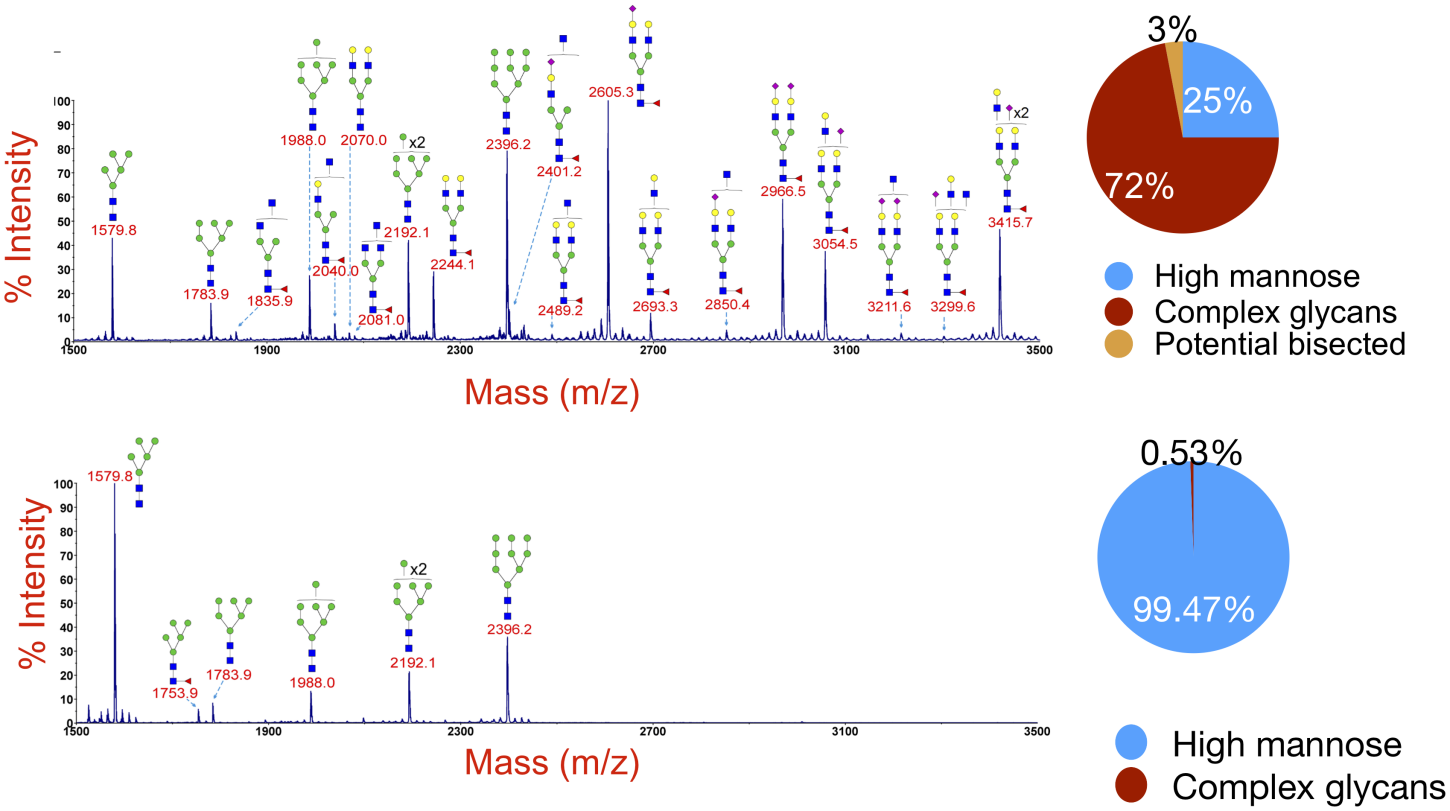
MALDI-TOF analysis of glycans present on gp120 produced by CHO-S and MGAT1^−^ CHO cell lines. The carbohydrates on purified A244 gp120s produced by CHO-S (A) and MGAT1^−^ CHO (B) cells were released by PNGase F digestion and examined by MALDI-TOF MS as described in the Materials and methods section. Pie charts indicate the percentage of high-mannose (blue), complex (red), and potential bisected (green) N-linked glycans. This analysis was performed by the Complex Carbohydrate Research Center at the University of Georgia. CHO, Chinese hamster ovary; MALDI-TOF, matrix-assisted laser desorption ionization–time of flight; MGAT1, Mannosyl (Alpha-1,3-)-Glycoprotein Beta-1,2-N-Acetylglucosaminyltransferase; MS, mass spectrometry; PNGase F, Peptide:N-Glycosidase F.

Additional studies were carried out to characterize the specific glycans incorporated in the A244-rgp120 produced in the MGAT1^−^ CHO and the CHO-S cell lines. Using MALDI-TOF-MS (Fig 6), we found that 56.4% of the N-linked glycans present on the MGAT1^−^ CHO–produced gp120 were Man_5_, 19.2% were Man_9_, 11% were Man_8_, and the remainder were Man_6_ and Man_7_. No complex, sialic acid–containing glycans were detected (Table 1). The degree of fucosylation was also significantly lowered; fucosylation was only on Man_5_ glycoforms at the core N-acetylglucosamine (GlcNAc) and represented 3.16% of the total glycans present.

**Table 1:** Percentage of different glycan species on gp120 produced by CHO-S and MGAT1^−^ CHO cells.

	Cell Line	
Glycan Species	CHO-S	MGAT1^−^ CHO
Man_5_	5.2%	56.4%
Man_6_	1.9%	4.5%
Man_7_	3.3%	7.1%
Man_8_	5.1%	11.4%
Man_9_	9.6%	19.2%
Complex or hybrid	75%	0.53%

Percentages calculated from MALDI-TOF peak intensity.

Abbreviations: CHO, Chinese hamster ovary; Man, mannose; MALDI-TOF, matrix-assisted laser desorption ionization–time of flight; MGAT1, Mannosyl (Alpha-1,3-)-Glycoprotein Beta-1,2-N-Acetylglucosaminyltransferase.

When the A244-rgp120 produced in CHO-S cells was examined, approximately 75% of the glycans were complex or hybrid glycans, and 25% represented the early intermediates ranging from Man_5_ to Man_9_. No high-mannose species were detected with core GlcNAc fucose attached, but nearly all hybrid and complex glycans were fucosylated.

### Binding multiple bN-mAbs to gp120 expressed in the MGAT1^−^ CHO cells

**Fig 7:**
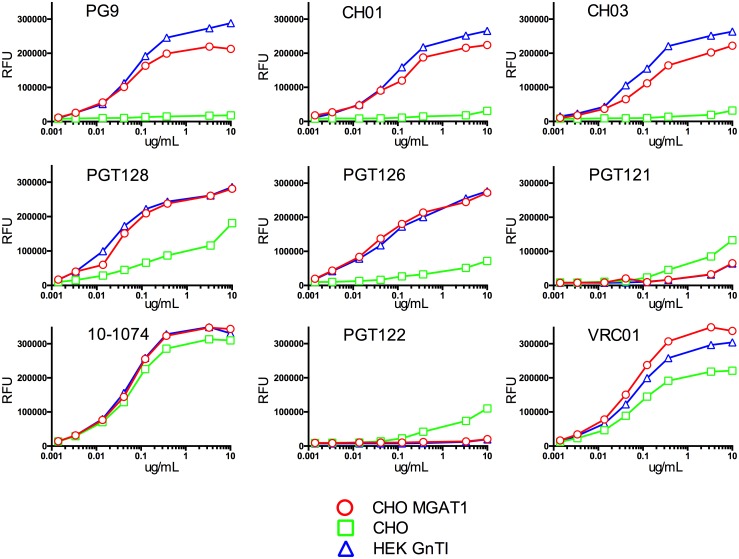
Comparison of bN-mAb binding to A244-rgp120 produced in MGAT1^−^ CHO cells, CHO-S cells, and HEK 293 GnTI^−^ cells. The binding of a panel of bN-mAbs to purified A244-rgp120 produced in MGAT1^−^ CHO cells, CHO-S cells, and HEK 293 GnTI^−^ cells was measured in an FIA. Briefly, purified proteins were captured onto wells of black 96-well microtiter plates coated with a mouse monoclonal antibody against the N-terminal gD tag present in all 3 proteins. Plates were then incubated with serial dilutions of bN-mAbs targeting glycan-epitopes within the V1V2 domain (PG9, CH01, and CH03), the glycan-epitopes within the V3 domain (PGT128, PGT126, PGT121, 10–1074, and PGT122), or the CD4 binding site (VRC01). Plates were incubated with a 1:3,000 dilution of AlexaFluor 488 conjugated goat-anti-human polyclonal antibody, and binding is reported as RFUs. FIA details are provided in the Materials and methods section. Underlaying data can be found in S2 Data. bN-mAb, broadly neutralizing monoclonal antibody; CHO, Chinese hamster ovary; FIA, fluorescence immunoassay; gD, glycoprotein D; GnTI, N-acetylglucosaminyltransferase I; HEK 293, human embryonic kidney 293; MGAT1, Mannosyl (Alpha-1,3-)-Glycoprotein Beta-1,2-N-Acetylglucosaminyltransferase; RFU, relative fluorescence unit; rgp120, recombinant gp120.

We next compared A244-rgp120 expressed in the MGAT1^−^ CHO cells with A244-rgp120 produced in normal CHO-S cells for the ability to bind bN-mAbs in an FIA. A panel of prototypic bN-mAbs that recognize distinct sites of virus vulnerability in monomeric and trimeric Envs was utilized (Fig 7). We noted a significant improvement in the binding of PG9, CH01, and CH03 to the proteins expressed in MGAT1^−^ CHO cells and HEK 293 GnTI^−^ cells compared to the CHO-S cells. These bN-mAbs are known to bind to epitopes in the V1/V2 domain that require Man_5_ at the N160 glycosylation site [3]. Similarly, we noted a significant improvement in the binding of the PGT126 and PGT128 bN-mAbs that require oligomannose glycans at the N301 and N332 glycosylation sites in the stem of the V3 domain [51]. Mixed results were seen for the PGT121 family of bN-mAbs in which the binding to gp120 produced in both the MGAT1^−^ CHO and HEK 293 GnTI^−^ cells lines was lower than binding of these antibodies to gp120 produced in the CHO-S cell line. In contrast, binding to the 10–1074 bN-mAb, also in the PGT121 family, was unaffected by the cellular substrate used for production. These results demonstrate that changing the glycosylation while leaving the amino acid sequence intact can significantly improve the antigenic structure of A244-rgp120 with respect to the binding of several bNAbs to glycan-dependent epitopes. The effect of differences in glycosylation was also examined on the binding of the VRC01 bN-mAb, known to recognize a glycan-independent epitope adjacent to the CD4 binding site [52]. While this antibody bound to all of the envelope proteins tested, a small but consistent improvement was observed in binding to the proteins produced in the MGAT1^−^ CHO and HEK 293 GnTI^−^ cells compared to the protein produced in the CHO-S cells. These studies suggest that the sialic acid–containing hybrid and complex carbohydrates incorporated in normal cell lines in some way interfere with the VRC01 binding site on monomeric gp120. This same effect may be observed with further non-glycan-dependent antibodies by decreasing the glycan interference.

### Minute virus of mice (MVM) infectivity

MVM is a small inactivation-resistant virus that is ubiquitous in the environment and a major cause of bioreactor culture failure in biopharmaceutical manufacturing [53]. As sialic acid is a major receptor for MVM infectivity, the MGAT1^−^ CHO cell line we created might have the additional manufacturing benefit of being resistant to MVM infection [54]. To investigate this possibility, the MGAT1^−^ CHO cell line was tested for infectivity resistance to 2 strains of MVM using a quantitative polymerase chain reaction (qPCR) assay and compared to wild-type CHO-S MVM sensitivity. While the MGAT1^−^ CHO cell line was similarly sensitive to the MVM prototypic strain (MVMp) as wild-type CHO-S cells, it was resistant to MVM Cutter strain (MVMc) infection (Table 2). The receptor proteins for MVM have not yet been identified, but it has been demonstrated that MVMp binds to sialic acid residues from both N- and O-linked glycosylation [55–57]. Knocking out MGAT1 does not alter the O-linked glycosylation pathway, perhaps explaining why the line remains sensitive to MVMp. MVMc is a more recently identified strain [58] with little information available on its binding to CHO or murine cells. Anything beyond noting the apparent dependence on complex N-linked glycosylation would be speculative at this point.

**Table 2:** MVM infectivity assay.

MVM type	Cell	MVM Cp	18s Cp	MVM Copies	18S Copies	MVM/18S	MVM/18S
MVMp	CHO-S	5.80	17.98	2.23E+10	2.03E+06	1.13E+04	1.13E+04
	MGAT1^−^ CHO	6.11	18.74	1.95E+10	1.22E+06	1.49E+04	1.49E+04
MVMc	CHO-S	7.91	18.91	5.04E+09	1.07E+06	4.84E+03	4.84E+03
	MGAT1^−^ CHO	19.3	19.6	2.00E+06	6.91E+05	2.92E+00	2.92E+00

All values are the mean of a triplicate set. Cp is the cycle at which fluorescence from amplification becomes greater than background and is used to infer copy number against a standard curve with a lower Cp.

Abbreviations: 18S, eukaryotic ribosomal subunit; CHO, Chinese hamster ovary; Cp, quantitative PCR crossover point; MGAT, MGAT1, Mannosyl (Alpha-1,3-)-Glycoprotein Beta-1,2-N-Acetylglucosaminyltransferase; MVMc, MVM Cutter strain; MVMp, MVM prototypic strain.

This infectivity assay was performed by IDEXX BioResearch (Columbia, Missouri, United States).

### Adventitious agent testing

The cell line was tested for the presence of mycoplasma, cross-species contamination, and viral contaminants by IDEXX BioResearch (Columbia, Missouri, US). No adventitious agents were detected. The full list and procedure are described in S1 and S2 Tables.

## Discussion

A major goal in HIV vaccine research is to develop immunogens that elicit bNAbs. The discovery that multiple bN-mAbs to HIV recognize glycan-dependent epitopes has altered our thinking of how best to produce this vaccine. Instead of using standard CHO cell production cell lines, which incorporate complex and hybrid glycosylation, a cell line that limits glycosylation to high-mannose forms may be useful for gp120 immunogens. While we have long been able to produce properly folded Env protein monomers (gp120 and gp140), as indicated by the ability to bind CD4 with high affinity [59–63], until now, we have not been able to replicate the glycan structures required for the binding of multiple families of bN-mAbs in expression systems suitable for large-scale manufacturing.

The glycans that decorate the surface of native, virion-associated Envs are typically enriched for high-mannose variants, normally found on early intermediate proteins within the endoplasmic reticulum (ER) and early Golgi [18,19]. This unusual restriction in glycan maturation is thought to be a consequence of steric hindrance occurring during the formation of trimeric virus spike structures [64]. Additionally, the high density of PNGSs that likely evolved as a glycan shield to prevent immune recognition of virus sequences [38,47,65] appears to limit glycosidase and glycosyltransferase modifications of Env glycans in the late ER and early Golgi apparatus (GA) [18,64]. While expression of monomeric gp120 results in incorporation of complex glycosylation, trimeric spike formation results in incomplete glycosylation and enrichment of virions with high-mannose glycans [18,19]. These differences in glycosylation might explain the inability of previous HIV vaccines to elicit bNAbs to glycan-dependent epitopes in humans. However, they do not explain the inability of previous vaccines to elicit bNAbs to epitopes such as VRC01 that were present in most gp120 vaccines expressed in normal CHO cells. Earlier vaccines such as the AIDSVAX B/E used in the RV144 trial largely possessed complex sialic acid–containing glycans and lacked the high-mannose glycans required for a variety of bN-mAbs, including PG9, CH01, CH03, PGT128, and 10–1074 [16]. Although the level of protection achieved in the 16,000-person RV144 trial was statistically significant (31.2%, *P* = 0.04), this level was not sufficient for product registration or clinical deployment. In this regard, the addition of 1 or more epitopes recognized by bN-mAbs, such as the PG9 and PGT128 epitopes described in this report, might improve the antigenic structure and immunogenicity of or recombinant gp120 such that a level of protection of 50% or more—which is required for product approval—is achieved. Currently, RV144 follow-up studies are in progress that make use of sialic acid–containing gp120 vaccine antigens produced in normal CHO cells, like those used in the original RV144 trial [66,67]. These new trials are trying to improve the level of vaccine efficacy by prolonging the immunization schedule, altering the adjuvant formulation, and replacing the canarypox vector coadministered with gp120 with stronger, more virulent virus vectors.

Few methods currently exist to produce recombinant proteins incorporating the Man_5_ and Man_9_ glycans that are present in the gp120 Env. Expression of gp120 in yeast results in the incorporation of long-chain high-mannose glycans [68], and insect expression systems produce a preponderance of paucimannose forms (Man_3-4_) [69]. Glycosidase inhibitors (e.g., kifunensine and swainsonine, see Fig 1) are effective and useful for producing analytical quantities of proteins with Man_5_ and Man_9_ intermediates but are highly toxic and prohibitively expensive for large-scale biopharmaceutical production [70,71]. Additionally, there exists evidence to suggest a broad mannosidase inhibitor like kifunensine may negatively impact protein folding through interference of the calnexin/calreticulin pathway [72–75]. Glycosaminyl-transferase knockout cell lines from 293 HEK and CHO cells, referred to as HEK 293 GnTI^−^ and CHO Lec1, respectively, have previously been described. They were generated through random ethyl methanesulfonate (EMS) mutagenesis, zinc finger methods, or screening for modified glycosylation by resistance to cytotoxic lectin binding [76–78]. These lines lack a functional MGAT1 gene, responsible for the protein GnTI. Knocking out the MGAT1 gene prevents processing of glycans beyond the Man_5_GlcNAc_2_ stage, resulting in exclusively high-mannose glycoprotein production [28,29]. Such cell lines do not generally grow as robustly as their parental counterparts, and they raise potential regulatory issues with potential uncharacterized genetic alterations. In light of this, there is an unmet need for a cell line suitable for the scalable production of HIV vaccine immunogens. We have addressed this problem by creating the novel MGAT1^−^ CHO cell line described above. Our data suggest that this cell line possesses several essential characteristics required for current Good Manufacturing Practices (cGMPs), such as robust growth in well-defined serum-free medium, the ability to grow to high cell densities in suspension culture, a well-defined mutation of the MGAT1 gene, and freedom from contamination by adventitious agents. However, the ultimate utility of this cell line will require characterization of stable cell lines with transfected envelope proteins in which the genetic stability of the transgene as well as the quality and yield of the final product is determined.

Recombinant envelope proteins produced in the MGAT1^−^ CHO cell line, such as the A244-rgp120 described in this report, can be used to test the hypothesis that previous HIV vaccines such as the AIDSVAX B/E vaccine used in the RV144 trial [12–14] were ineffective because they lacked the glycan-dependent epitopes required to stimulate the formation of bN-mAbs. While the CHO MGAT1^−^ cell line provides a practical way to produce envelope proteins with several glycan-dependent epitopes recognized by bNAbs not present on gp120s produced in normal CHO cell lines, we do not yet know whether these epitopes will be immunogenic. Previous gp120 vaccine trials such as RV144 failed to detect glycan-independent VRC01-like antibodies even though the VRC01 epitope was present on at least 2 different gp120s used for immunization. Thus, some epitopes recognized by bNAbs are poorly immunogenic, and additional immunogenicity and formulation studies will likely be required to optimize virus-neutralizing antibody responses to the glycan epitopes of the type described in this paper. Although the gp120 expression data presented here were derived exclusively from small-scale transient transfection experiments, we anticipate that the cell line described in this report will be useful for the development of stable MGAT1^−^ CHO cell lines producing vaccines based on a variety of new concepts. These include guided immunization to stimulate germline genes encoding bNAbs [79–81], Env proteins designed with features that enhance antigen processing and presentation [82], and glycopeptide scaffolds that enhance the immunogenicity of epitopes recognized by bN-mAbs while eliminating nonprotective immunodominant epitopes [21].

## Materials and methods

### Cell culture

Suspension-adapted CHO-S cells were obtained from Thermo Fisher (Thermo Fisher, Life Technologies, Carlsbad, CA, US). HEK 293 GnTI^−^ suspension-adapted cells were obtained from ATCC (ATCC, Manassas, VA, US). Stocks of suspension-adapted CHO-S and HEK 293 GnTI^−^ cells were maintained in shake flasks (Corning, Corning, NY, US) using a Kuhner ISF1-X shaker incubator (Kuhner, Birsfelden, Switzerland). For cell propagation, shake flask cultures were maintained at 37 °C, 8% CO_2_, and 125 rpm. Static cultures were maintained in 96- or 24-well cell culture dishes and grown in a Sanyo incubator (Sanyo, Moriguchi, Osaka, Japan) at 37 °C and 8% CO_2_.

CHO-S cells were maintained in CD-CHO medium supplemented with 0.1% pluronic acid, 8 mM GlutaMax, and 1X Hypoxanthine/Thymidine (Thermo Fisher, Life Technologies, Carlsbad, CA, US). For cell growth studies, CHO cells were grown in BalnCD CHO Growth A Medium (Irving Scientific, Santa Ana, CA, US). HEK 293 GnTI^−^ cells were maintained in Freestyle 293 cell culture media (Life Technologies, Carlsbad, CA, US). During transient CHO cell protein production, the cells were maintained in OptiCHO medium supplemented with 0.1% pluronic acid, 2 mM GlutaMax, and 1X H/T (Thermo Fisher, Life Technologies, Carlsbad, CA, US). For protein production experiments, the growth medium was supplemented with CHO Growth A (Molecular Devices, Sunnyvale, CA, US), 0.5% Yeastolate (BD, Franklin Lakes, NJ, US), 2.5% CHO-CD Efficient Feed A, and 0.25 mM GlutaMax, 2 g/L Glucose (Sigma-Aldrich St. Louis, MO, US). Cell counts were performed using a TC20 automated cell counter (BioRad, Hercules, CA, US) with viability determined by trypan blue (Thermo Fisher, Life Technologies, Carlsbad, CA, US) exclusion. Cell-doubling time in hours was calculated using the formula ((T_2_ − T_1_) × log_2_) / (log(D_2_) − log(D_1_)), where T = time at count and D = density at count. Cell count numbers used for doubling time calculation were from the logarithmic phase of growth.

### Gene sequencing

The sequence of the MGAT1 CHO gene was confirmed using primers based on the predicted mRNA transcript (XM_007644560.1 [83]). Genomic DNA was extracted using the AllPrep kit (Qiagen, Germantown, MD, US). The MGAT1 gene was PCR amplified using the primers F_CAGGCAAGCCAAAGGCAGCCTTG and R_CTCAGGGACTGCAGGCCTGTCTC (Eurofins Genomics, Louisville, KY, US) with Taq and dNTPs supplied by New England BioLabs (Ipswich, MA, US). The PCR product was gel purified using a Zymoclean kit (Zymo Research, Irvine, CA, US) and then sequenced by Sanger method at UC Berkeley, Berkeley, CA, US. MGAT1 knockouts were sequenced in the same manner.

### CRISPR/Cas9 target design and plasmid preparation

We utilized a CRISPR/Cas9 nuclease vector with an OFP reporter (GeneArt, Thermo Fischer Scientific, Waltham, MA, US). Three target sequences to knock out the CHO-S MGAT1 gene were designed using an online CRISPR RNA Configurator tool (GE Dharmacon, Lafayette, CO, US): Target 1: CCCTGGAACTTGCGGTGGTC; Target 2: GGGCATTCCAGCCCACAAAG; Target 3: GGCGGAACACCTCACGGGTG. Each sequence was run in NCBI’s BLAST tool for homologies with off-target sites in the CHO genome. Single-stranded DNA oligonucleotides and their complement strands were synthesized (Eurofins Genomics, Louisville, KY, US) with extra bases on the 3′ ends for ligation into a GeneArt CRISPR nuclease vector (Thermo Fisher, GeneArt, Waltham, MA, US). The strands were ligated and annealed into a GeneArt CRISPR vector using the protocol and reagents supplied with the kit. One Shot TOP10 Chemically Competent *Escherichia coli* were transformed and plated following the Invitrogen protocol (Thermo Fisher, Invitrogen, Carlsbad, CA, US). These were incubated in 5 mL LB broth at 37 °C in a shaking incubator at 225 rpm overnight. Minipreps were performed according to manufactures instructions (Qiagen, Germantown, MD, US) and sent to UC Berkeley DNA Sequencing Facility (Berkeley, CA, US) with the U6 primers included in the GeneArt CRISPR kit to confirm successful integration of guide sequences. A single 500-mL Maxiprep was performed for each of the 3 target sequences using PureLink Maxiprep kit (Thermo Fisher, Invitrogen, Carlsbad, CA, US).

### Electroporation

Electroporation of CHO cells was performed using a MaxCyte STX scalable transfection system (MaxCyte, Gaithersburg, MD, US) according to the manufacturer’s instructions. Briefly, CHO-S cells were maintained at >95% viability prior to transfection. Cells were pelleted at 250 g for 10 minutes and then resuspended in MaxCyte EP buffer (MaxCyte, Gaithersburg, MD, US) at a density of 2 × 10^8^ cells/mL. Transfections were carried out in the OC-400 processing assembly (MaxCyte, Gaithersburg, MD, US) with a total volume of 400 μL and 8 × 10^7^ total cells. CRISPR/Cas9 exonuclease with guide sequence plasmid DNA suspended in endotoxin-free water was added to the cells in EP buffer for a 300-μg final concentration of DNA/mL. The processing assemblies were then transferred to the MaxCyte STX electroporation device, and the CHO protocol was selected using the MaxCyte STX software. Following electroporation, the cells in electroporation buffer were removed from the processing assembly and placed in 125-mL Erlenmeyer cell culture shake flasks (Corning, Corning, NY, US). The flasks were placed into 37 °C incubators with no agitation for 40 minutes. Following the rest period, prewarmed OPTI-CHO media were added to the flasks for a final cell density of 4 × 10^6^ cells/mL. Flasks were then moved to a Kuhner shaker and agitated at 125 rpm.

### Plating, expansion, and culture of CRISPR-transfected CHO-S cells

Twenty-four hours post transfection, a 100-μL aliquot was taken from each of the transfected pools to assay for cell viability and OFP expression using a light microscope (Zeiss Axioskop 2, Zeiss, Jena, Germany). Ninety-six-well flat-bottom cell culture plates (Corning, Corning, NY, US) were filled with 50 μL of conditioned CD-CHO media. Each of the 3 transfected pools was serially diluted with warmed media to 10 cells/mL and added to 5 plates per pool in 50-μL volumes. Final calculated cell density was 0.5 cells/well in 100 μL of media. Once any single-colony well reached approximately 20% confluency, the contents were transferred to a 24-well cell culture plate (Corning, Corning, NY, US) with 500 μL of fresh media. When confluency reached 50%, a 200-μL aliquot was removed for testing via a GNA-binding assay. Following positive lectin binding, cells were moved to a 6-well cell culture plate (Corning, Corning, NY, US) with 2 mL of media per well. After 5 days of growth in 6-well plates, the GNA assay was repeated. Colonies exhibiting positive lectin binding were moved to 125-mL shake flasks with an initial 6 mL of media. Daily counts were taken, and cell cultures were expanded to maintain a density of 0.3 × 10^6^−1.0 × 10^6^ cells/mL.

### Lectin binding assay

Fluorescein-labeled GNA, from the snowdrop pea (Vector Laboratories, Burlingame, CA, US), was used to detect the cell surface expression of Man_5_ glycoforms. Cell aliquots (200 μL) from 24-well plates were pelleted at 3,000 rpm for 3 minutes. The supernatant was discarded, and the cell pellet was washed 3 times with 500 μL of ice-cold 10 μM EDTA in (Boston BioProducts, Ashland, MA, US) phosphate-buffered saline (PBS) (Thermo Fisher, Gibco, Carlsbad, CA, US). The cell pellet was then resuspended in 200 μL of ice-cold 10 μM EDTA with PBS with 5 μg/mL of GNA-fluorescein. Samples were shielded from light and incubated on ice with GNA for 30 minutes. Following incubation, samples were washed 3 times and resuspended to a volume of 50 μl in 10 μM EDTA PBS. Samples were then examined under a light microscope (Zeiss Axioskop 2, Zeiss, Jena, Germany) with 495-nm wavelength excitation. Wild-type CHO-S cells were used as a negative control, and HEK 293 GnTI^−^ cells were used as a positive control. Representative images were taken on a Leica DM5500 B Widefield Microscope (Leica Microsystems, Buffalo Grove, IL, US) at the UC Santa Cruz microscopy center.

### Experimental protein production

An expression plasmid containing the gene encoding gp120 from the A244 strain of HIV (Genbank accession number: MG189369) was selected for transient transfection experiments. The protein encoded by this gene was identical to that used to produce the AIDSVAX B/E vaccine used in the RV144 trials [13,14], with the exception that the N-linked glycosylation site at N334 was moved to N332. For analytical scale experiments, a total of 4 × 10^5^ cells from each candidate MGAT1^−^ CHO line were placed in 450 μl of media in a 24-well cell culture plate. Fugene, 1.7 μL (Promega, Madison, WI, US), was preincubated at room temperature for 30 minutes with 550 ng of DNA in a total volume of 50 μL of media. Then, 50 μL of Fugene/DNA mixture was added to each well for a final transfected volume of 500 μL. Aliquots of supernatant were removed for assay 72 hours post transfection.

For preparative-scale transient transfection experiments, CHO cells were electroporated following the above MaxCyte method. Twenty-four hours post electroporation, the culture was supplemented with 1 mM sodium butyrate (Thermo Fisher, Life Technologies, Carlsbad, CA, US), and the temperature was lowered to 32 °C. The cultures were fed daily the equivalent of 3.5% of the original volume with Molecular Devices CHO A Feed (Molecular Devices, Sunnyvale, CA, US), 0.5% Yeastolate (BD, Franklin Lakes, NJ, US), 2.5% CHO-CD Efficient Feed A, and 0.25 mM GlutaMax, 2 g/L Glucose (Sigma-Aldrich St. Louis, MO, US). Cultures were run until cell viability dropped below 50%. Supernatant was harvested by pelleting the cells at 250 g for 30 minutes, followed by prefiltration through Nalgene Glass Prefilters (Thermo Scientific, Waltham, MA, US) and 0.45-μm SFCA filtration (Nalgene, Thermo Scientific, Waltham, MA, US) and then stored frozen at −20 °C until purification. Proteins were purified using an N-terminal affinity tag derived from type 1 herpes simplex virus glycoprotein D (gD) as previously described [16].

### Glycosidase digestion and SDS-PAGE

Endo H and PNGase F (New England BioLabs, Ipswich, MA, US) digests were performed per the manufacturer’s protocol on 5 μg of purified envelope protein using 1 unit of glycosidase. Samples were reduced, denatured, and then digested overnight at 37 °C. Digested samples were run on NuPAGE (Thermo Fisher, Invitrogen, Carlsbad, CA, US) 4%–12% BisTris precast gels in MES running buffer and then stained with SimplyBlue stain (Thermo Fisher, Invitrogen, Carlsbad, CA, US).

### Fluorescence immunoassays (FIAs) to measure antibody binding

A FIA was used to measure the binding of polyclonal or monoclonal antibodies to recombinant envelope proteins. For antibody binding to purified proteins, Greiner Fluortrac 600 microtiter plates (Greiner Bio One, Kremsmünster, Austria) were coated with 2 μg/mL of purified envelope protein overnight in PBS with shaking. Plates were blocked in PBS + 2.5% BSA (blocking buffer for 90 minutes and then washed 4 times with PBS containing 0.05% Tween-20 [Sigma]). Serial dilutions of monoclonal antibodies were added in a range from 10 μg/mL to 0.0001 μg/mL and then incubated at 25 °C for 90 minutes with shaking. After incubation and washing, 488 Alexa Fluor conjugated anti-human or anti-murine (Invitrogen, CA, US) was added at a 1:3,000 dilution in PBS + 1% BSA. Plates were incubated for 90 minutes with shaking and then washed 4 times with 0.05% Tween PBS using an automated plate washer. Plates were then imaged in a plate spectrophotometer (Envision System, Perkin Elmer) at excitation and emission wavelengths of 395 nm and 490 nm, respectively. For antibody binding to unpurified envelope proteins in cell culture supernatants, Greiner Fluortrac 600 microtiter plates (Greiner Bio-one, Germany) were coated with 2 μg/mL of purified mouse monoclonal antibody to an epitope in the V2 domain (10C10) or the gD purification tag (34.1) overnight in PBS with shaking. Plates were blocked in PBS + 2.5% BSA blocking buffer for 90 minutes and then washed 4 times with PBS containing 0.05% Tween-20 (Sigma). Then, 150 μl of 40× diluted supernatant was added to each well, or 10 μg/mL of purified protein in control lanes, and incubated at 25 °C for 90 minutes with shaking. After incubation and washing, PG9 was added in a range from 10 μg/mL to 0.0001 μg/mL and then incubated at 25 °C for 90 minutes with shaking. After incubation and washing, fluorescently conjugated anti-human or anti-murine (Invitrogen, CA, US) was added at a 1:3,000 dilution. Plates were incubated for 90 minutes with shaking and then washed 4 times with 0.05% Tween PBS using an automated plate washer. Plates were then imaged in a plate spectrophotometer (Envision System, Perkin Elmer, Waltham, MA, US) at excitation and emission wavelengths of 395 nm and 490 nm, respectively.

The bN-mAb PG9 was purchased from Polymun (Klosterneuburg, Austria) or produced in-house using 293 HEK cells from a synthetic gene created on the basis of published sequence data [84] (also available for purchase from the NIH AIDS Reagent Program, Germantown, MD, US. Catalog Number 12149). Alexa Fluor 488 conjugated anti-human IgG, anti-rabbit IgG, and anti-mouse IgG polyclonal antibodies were obtained from Invitrogen (Invitrogen, Thermo Fisher, Carlsbad, CA, US).

### Glycan composition analysis by MALDI-TOF-MS

A glycoprotein sample (approximately 100 μg) suspended in 50 mM ammonium bicarbonate buffer was incubated with trypsin (5 μg, Sigma Aldrich) for 18 hours at 37 °C. Digested peptides were desalted and purified by passing through a C18 sep-pak cartridge after inactivating trypsin by heating at 95 °C for 5 minutes. The purified peptides were then treated with PNGase F (23 IUB milliunits, NEB#P0705, New England BioLabs, Ipswich, MA) at 37 °C for 16 hours to release the N-glycans. The released N-glycans were desalted and purified from the peptides by C18 sep-pak cartridge, followed by freeze-drying. Finally, the N-glycans were permethylated for 10 minutes at room temperature by using 100 μl of methyl iodide in the presence of NaOH/DMSO base (350 μl). The reaction was quenched by adding water (1 mL), and the permethylated N-glycans were extracted by organic phase separation using dichloromethane (2 mL). The excess of dichloromethane was removed by stream of nitrogen and subsequently prepared for MALDI-MS analysis [84].

Permethylated N-glycans were dissolved in methanol (20 μl), and small aliquot (approximately 1 μl) was spotted on to a MALDI plate (Opti-TOF-384 well insert, Applied Biosystems, Foster City, CA, US) and crystallized with DHB matrix (20 mg/mL in 50% methanol/water, Sigma Aldrich). Data were obtained from AB SCIEX MALDI TOF/TOF 5800 (Applied Biosystem MDS Analytical Technologies, Foster City, CA, US) mass spectrometer in reflector positive-ion mode. Data analysis was performed by using Data Explorer V4.5, and the assignment of glycan structure was based on the primary mass (m/z) coupled with an MS/MS fragmentation profile using the Expasy database online and the glycowork bench software analysis [85,86]

### MVM infectivity assay and sterility testing

IDEXX BioResearch (Columbia, MO, US) performed an MVM infectivity assay. Cells were cultured at 4 × 10^5^ cells/mL in 100-mL total volume, under conditions described above, in a spinner flask for 5 days. CHO-S and MGAT1^−^ CHO cells were infected with 1 multiplicity of infection (MOI) of MVMp or MVMc and evaluated in triplicate. Five-mL aliquots were removed on days 1, 3, and 5, and cells were pelleted by centrifugation and stored at −20 °C. Day 5 samples were evaluated by qPCR for MVM and 18S using proprietary primers. The qPCR crossing point (CP) values were reported, and copy numbers were based upon standard curves.

The cell line was tested for a panel of adventitious agents, cell line species, and in vitro virus contamination by IDEXX BioResearch (Columbia, MO, US) using a PCR-based protocol described in S1 Text.

## Supporting information

S1 Data Underlaying FIA data for initial line selection. Data from this table, generated as described in the Materials and methods section, were used to create the plot shown in Figs 2F and 4A using Prism 6 software. FIA, fluorescence immunoassay.
(DOCX)

S2 Data Underlaying FIA data for initial line selection. Data from this table, generated as described in the Materials and methods section, were used to create the plots shown in Fig 7 using Prism 6 software. FIA, fluorescence immunoassay.
(DOCX)

S1 Table Cross-species contamination assay. Real-time PCR was performed by IDEXX BioResearch to confirm the absence of mammalian cross-species contamination. The presence of cross-species contaminants is indicated by a “+.” A negative finding is indicated by “-”.
(DOCX)

S2 Table Pathogenic agent contamination test results. IDEXX laboratories (Columbia, Missouri, US) performed real-time PCR to detect whether any pathogenic agents were present in the MGAT1 CHO cell line. This followed IDEXX’s IMPACT2F and h-IMPACT Profile 1 profile of tests. A “+” indicates a positive, and “-”indicates a negative result. Not shown are positive and negative control results. These were performed using low copy numbers of synthetic oligos corresponding to the tested-for sequences (positive) and primer-free reactions (negative). CHO, Chinese hamster ovary; MGAT1, Mannosyl (Alpha-1,3-)-Glycoprotein Beta-1,2-N-Acetylglucosaminyltransferase.
(DOCX)

S1 Text IDEXX PCR methodology. (DOCX)

## Abbreviations

18S: eukaryotic ribosomal subunit
bNAb: broadly neutralizing antibody
bN-mAb: broadly neutralizing monoclonal antibody
Cas9: CRISPR-associated protein 9
cGMP: current Good Manufacturing Practice
CHO: Chinese hamster ovary
Cp: qPCR crossing point
CRISPR: clustered regularly interspaced palindromic repeat
DIC: differential interference contrast
DSB: double-stranded break
EMS: ethyl methanesulfonate
Endo H: endoglycosidase H
Env: HIV envelope protein
ER: endoplasmic reticulum
FIA: fluorescence immunoassay
GA: Golgi apparatus
gD: glycoprotein D
GlcNAc: N-acetylglucosamine
GNA: *Galanthus nivalis* lectin
GnTI: N-acetylglucosaminyltransferase
gRNA: guide RNA
HDR: homology-directed repair
HEK 293: human embryonic kidney 293
MALDI-TOF-MS: matrix-assisted laser desorption ionization-time of flight mass spectrometry
Man_5_: mannose-5
Man_9_: mannose-9
MGAT1: Mannosyl (Alpha-1,3-)-Glycoprotein Beta-1,2-N-Acetylglucosaminyltransferase
MOI: multiplicity of infection
MVM: minute virus of mice
MVMc: MVM Cutter strain
MVMp: MVM prototypic strain
NHEJ: nonhomologous end joining
NHEJR: nonhomologous end joining repair
N-X-S/T: asparagine-X-serine/threonine
OFP: orange fluorescent protein
PAM: protospacer-adjacent motif
PBS: phosphate-buffered saline
PNGase F: Peptide:N-Glycosidase F
PNGS: potential N-linked glycosylation site
qPCR: quantitative polymerase chain reaction
RFU: relative fluorescence unit
rgp120: recombinant gp120
tracrRNA: *trans*-activating CRISPR RNA
