# Supplementary material for: CRISPR/Cas9 gene editing for the creation of an MGAT1-deficient CHO cell line to control HIV-1 vaccine glycosylation
Source: PLoS Biol. 2018 Aug 29;16(8):e2005817. doi: 10.1371/journal.pbio.2005817 (PMC6133382; doi:10.1371/journal.pbio.2005817)
Supplement: S1 Data — Data from this table, generated as described in the Materials and methods section, were used to create the plot shown in Figs 2F and 4A using Prism 6 software. FIA, fluorescence immunoassay. (DOCX) [file pbio.2005817.s001.docx]

| **PG9 ug/mL** | **3.5D9** | **3.5D9** | **3.5D8** | **3.5D8** | **3.4F10** | **3.4F10** | **3.5A2** | **3.5A2** | **CHO-S** | **CHO-S** | **GnTI** | **GnTI** |
| --- | --- | --- | --- | --- | --- | --- | --- | --- | --- | --- | --- | --- |
| **10** | 43597 | 46993 | 61432 | 60958 | 81197 | 114405 | 57549 | 57406 | 14154 | 14552 | 91713 | 89892 |
| **3.33** | 29150 | 35466 | 46708 | 42793 | 64009 | 61419 | 44078 | 46094 | 11637 | 11680 | 67870 | 66376 |
| **1** | 21646 | 23894 | 34856 | 32514 | 46625 | 47884 | 32427 | 33494 | 11220 | 11452 | 48277 | 48356 |
| **0.33** | 16951 | 16707 | 22067 | 21662 | 31647 | 30209 | 20757 | 23384 | 10171 | 10531 | 31552 | 32513 |
| **0.1** | 14378 | 13162 | 15867 | 16712 | 19730 | 19597 | 16585 | 15934 | 8776 | 10273 | 18031 | 20551 |
| **0.033** | 11101 | 11329 | 11622 | 12859 | 14412 | 13110 | 12579 | 11925 | 8725 | 8102 | 12654 | 14438 |
| **0.01** | 9671 | 10468 | 9830 | 10254 | 11588 | 10772 | 9954 | 10459 | 9500 | 9736 | 11199 | 10725 |
| **0.001** | 8178 | 8184 | 8229 | 8159 | 10403 | 9279 | 10278 | 12115 | 9400 | 9216 | 9800 | 9824 |
