# Supplementary material for: CRISPR/Cas9 gene editing for the creation of an MGAT1-deficient CHO cell line to control HIV-1 vaccine glycosylation
Source: PLoS Biol. 2018 Aug 29;16(8):e2005817. doi: 10.1371/journal.pbio.2005817 (PMC6133382; doi:10.1371/journal.pbio.2005817)
Supplement: S2 Data — Data from this table, generated as described in the Materials and methods section, were used to create the plots shown in Fig 7 using Prism 6 software. FIA, fluorescence immunoassay. (DOCX) [file pbio.2005817.s002.docx]

| **PG9 ug/mL** | **CHO MGAT1** | **CHO MGAT1** | **CHO-S** | **CHO-S** | **HEK GntI** | **HEK GntI** |
| --- | --- | --- | --- | --- | --- | --- |
| **10** | 198819 | 226538 | 15972 | 20426 | 285659 | 290863 |
| **3.333** | 196873 | 242058 | 15225 | 19200 | 262196 | 284780 |
| **0.37** | 178388 | 219710 | 13364 | 16930 | 244551 | 246899 |
| **0.123456** | 150200 | 176790 | 12175 | 14580 | 193180 | 190824 |
| **0.0411** | 93935 | 108555 | 9726 | 11707 | 118533 | 106912 |
| **0.0137** | 53895 | 58209 | 10530 | 9972 | 43380 | 59608 |
| **0.0034** | 22819 | 28943 | 9842 | 8243 | 25401 | 26234 |
| **0.0014** | 13611 | 10261 | 8904 | 7586 | 12787 | 7289 |
| **CH01 ug/mL** | **CHO MGAT1** | **CHO MGAT1** | **CHO-S** | **CHO-S** | **HEK GntI** | **HEK GntI** |
| **10** | 185579 | 262784 | 20336 | 41634 | 252798 | 278433 |
| **3.333** | 193882 | 238182 | 18356 | 17698 | 252265 | 251599 |
| **0.37** | 171887 | 203572 | 14746 | 14848 | 226270 | 209355 |
| **0.123456** | 128660 | 110728 | 11362 | 10876 | 174223 | 143692 |
| **0.0411** | 93236 | 87144 | 9479 | 8623 | 112281 | 75990 |
| **0.0137** | 47725 | 46990 | 9008 | 8249 | 60689 | 38479 |
| **0.0034** | 21172 | 32528 | 9510 | 8263 | 23748 | 22089 |
| **0.0014** | 11977 | 24155 | 8605 | 7224 | 11928 | 11180 |
| **CH03 ug/mL** | **CHO MGAT1** | **CHO MGAT1** | **CHO-S** | **CHO-S** | **HEK GntI** | **HEK GntI** |
| **10** | 204281 | 239892 | 43735 | 20236 | 260328 | 266578 |
| **3.333** | 190134 | 214532 | 24862 | 13915 | 254919 | 248102 |
| **0.37** | 162900 | 165901 | 16560 | 10898 | 235991 | 205751 |
| **0.123456** | 125498 | 98495 | 11851 | 8325 | 179734 | 130407 |
| **0.0411** | 82079 | 48560 | 10013 | 8968 | 143537 | 68256 |
| **0.0137** | 47344 | 26631 | 9836 | 8458 | 54845 | 32048 |
| **0.0034** | 21117 | 14909 | 8580 | 7192 | 29887 | 16690 |
| **0.0014** | 11296 | 9618 | 8322 | 8181 | 14215 | 15703 |
| **PGT128 ug/mL** | **CHO MGAT1** | **CHO MGAT1** | **CHO-S** | **CHO-S** | **HEK GntI** | **HEK GntI** |
| **10** | 238404 | 323623 | 100805 | 261273 | 269406 | 302673 |
| **3.333** | 216328 | 304835 | 72753 | 158938 | 226968 | 295820 |
| **0.37** | 200793 | 274672 | 63486 | 110526 | 221852 | 265627 |
| **0.123456** | 175891 | 242965 | 49663 | 82485 | 200462 | 243858 |
| **0.0411** | 127839 | 174360 | 35325 | 54801 | 157648 | 188100 |
| **0.0137** | 83177 | 36752 | 22463 | 35173 | 86550 | 112677 |
| **0.0034** | 34887 | 45459 | 11503 | 19011 | 33126 | 47776 |
| **0.0014** | 14728 | 19208 | 8883 | 11070 | 13870 | 18660 |
| **PGT126 ug/mL** | **CHO MGAT1** | **CHO MGAT1** | **CHO-S** | **CHO-S** | **HEK GntI** | **HEK GntI** |
| **10** | 244961 | 298959 | 64700 | 78892 | 269809 | 282754 |
| **3.333** | 217369 | 271705 | 43759 | 59148 | 259099 | 252249 |
| **0.37** | 184392 | 243274 | 28373 | 36719 | 191919 | 208942 |
| **0.123456** | 155400 | 205241 | 26565 | 26638 | 163079 | 181903 |
| **0.0411** | 118548 | 156309 | 16527 | 16289 | 111045 | 124393 |
| **0.0137** | 67636 | 100250 | 12070 | 13715 | 65823 | 91225 |
| **0.0034** | 29921 | 57204 | 10342 | 10163 | 29317 | 54123 |
| **0.0014** | 16927 | 22543 | 11066 | 9069 | 15169 | 20548 |
| **PGT121 ug/mL** | **CHO MGAT1** | **CHO MGAT1** | **CHO-S** | **CHO-S** | **HEK GntI** | **HEK GntI** |
| **10** | 62560 | 68035 | 118352 | 147524 | 55378 | 73008 |
| **3.333** | 31602 | 34142 | 77955 | 92132 | 28258 | 35525 |
| **0.37** | 17221 | 16164 | 43590 | 47682 | 15139 | 17944 |
| **0.123456** | 8897 | 10567 | 22974 | 23947 | 10101 | 11696 |
| **0.0411** | 23117 | 18141 | 12241 | 13540 | 8834 | 8522 |
| **0.0137** | 8561 | 8494 | 11458 | 10676 | 8284 | 7228 |
| **0.0034** | 8383 | 7568 | 8937 | 8321 | 9135 | 6633 |
| **0.0014** | 7983 | 7714 | 9623 | 8020 | 8406 | 6962 |
| **10-1074 ug/mL** | **CHO MGAT1** | **CHO MGAT1** | **CHO-S** | **CHO-S** | **HEK GntI** | **HEK GntI** |
| **10** | 350027 | 337092 | 289964 | 330047 | 331382 | 329930 |
| **3.333** | 355504 | 339101 | 302371 | 323645 | 362234 | 335980 |
| **0.37** | 331458 | 315302 | 281226 | 290360 | 338275 | 317641 |
| **0.123456** | 271281 | 239137 | 231541 | 219385 | 272261 | 245136 |
| **0.0411** | 150947 | 137414 | 127517 | 129750 | 163145 | 148387 |
| **0.0137** | 78793 | 74246 | 68142 | 72226 | 79215 | 79595 |
| **0.0034** | 29319 | 33101 | 25923 | 32087 | 28128 | 33939 |
| **0.0014** | 14101 | 14274 | 13237 | 13783 | 11836 | 14092 |
| **PGT122 ug/mL** | **CHO MGAT1** | **CHO MGAT1** | **CHO-S** | **CHO-S** | **HEK GntI** | **HEK GntI** |
| **10** | 19014 | 21387 | 87548 | 132623 | 16942 | 21685 |
| **3.333** | 12283 | 14928 | 60475 | 86647 | 11206 | 13030 |
| **0.37** | 11319 | 12477 | 37648 | 45952 | 8237 | 8797 |
| **0.123456** | 11129 | 9846 | 20033 | 25240 | 7884 | 7392 |
| **0.0411** | 11336 | 8914 | 13411 | 14299 | 7694 | 7960 |
| **0.0137** | 11988 | 8856 | 10208 | 10464 | 7805 | 7581 |
| **0.0034** | 12208 | 4744 | 8950 | 10805 | 7957 | 6722 |
| **0.0014** | 9276 | 9221 | 8860 | 8891 | 7681 | 7561 |
| **VRCO1 ug/mL** | **CHO MGAT1** | **CHO MGAT1** | **CHO-S** | **CHO-S** | **HEK GntI** | **HEK GntI** |
| **10** | 317315 | 357696 | 176404 | 265565 | 284136 | 324011 |
| **3.333** | 338967 | 358082 | 187131 | 249553 | 282645 | 310072 |
| **0.37** | 295229 | 318916 | 166896 | 215960 | 242256 | 273749 |
| **0.123456** | 212542 | 262731 | 122860 | 167082 | 176931 | 221680 |
| **0.0411** | 122435 | 178598 | 69535 | 108346 | 101731 | 142921 |
| **0.0137** | 60401 | 95924 | 36940 | 56378 | 50039 | 81688 |
| **0.0034** | 23822 | 44955 | 16828 | 29761 | 20455 | 39859 |
| **0.0014** | 13785 | 19065 | 11244 | 13033 | 11286 | 17739 |
