# Supplementary material for: CRISPR/Cas9 gene editing for the creation of an MGAT1-deficient CHO cell line to control HIV-1 vaccine glycosylation
Source: PLoS Biol. 2018 Aug 29;16(8):e2005817. doi: 10.1371/journal.pbio.2005817 (PMC6133382; doi:10.1371/journal.pbio.2005817)
Supplement: S1 Table — Real-time PCR was performed by IDEXX BioResearch to confirm the absence of mammalian cross-species contamination. The presence of cross-species contaminants is indicated by a “+.” A negative finding is indicated by “-”. (DOCX) [file pbio.2005817.s003.docx]

| Species | Result |
| --- | --- |
| Mouse | - |
| Rat | - |
| Human | - |
| Chinese hamster | + |
| African green monkey | - |
