# Supplementary material for: CRISPR/Cas9 gene editing for the creation of an MGAT1-deficient CHO cell line to control HIV-1 vaccine glycosylation
Source: PLoS Biol. 2018 Aug 29;16(8):e2005817. doi: 10.1371/journal.pbio.2005817 (PMC6133382; doi:10.1371/journal.pbio.2005817)
Supplement: S2 Table — IDEXX laboratories (Columbia, Missouri, US) performed real-time PCR to detect whether any pathogenic agents were present in the MGAT1 CHO cell line. This followed IDEXX’s IMPACT2F and h-IMPACT Profile 1 profile of tests. A “+” indicates a positive, and “-”indicates a negative result. Not shown are positive and negative control results. These were performed using low copy numbers of synthetic oligos corresponding to the tested-for sequences (positive) and primer-free reactions (negative). CHO, Chinese hamster ovary; MGAT1, Mannosyl (Alpha-1,3-)-Glycoprotein Beta-1,2-N-Acetylglucosaminyltransferase. (DOCX) [file pbio.2005817.s004.docx]

| Pathogen | Result | Pathogen | Result |
| --- | --- | --- | --- |
| EBV | - | MAV1 | - |
| HAdV | - | MAV2 | - |
| HCMV | - | mCMV | - |
| Hepatitis A | - | MHV | - |
| Hepatitis B | - | MNV | - |
| Hepatitis C | - | MTV | - |
| HHV 6 | - | *Mycoplasma pulmonis* | - |
| HHV 8 | - | *Mycoplasma sp.* | - |
| HIV1 | - | MVM | - |
| HIV2 | - | MPV | - |
| HPV16 | - | KRV | - |
| HPV18 | - | RMV | - |
| HSV 1 | - | RPV | - |
| HSV 2 | - | H1 | - |
| HTLV 1 | - | Polyoma | - |
| HTLV 2 | - | PVM | - |
| VZV | - | rCMV | - |
| Ectromelia | - | RCV/SDAV | - |
| EDIM | - | REO3 | - |
| Hantaan | - | Sendai | - |
| K virus | - | Seoul | - |
| LCMV | - | Sin Nombre | - |
| LDEV | - | Treponema pallidum | - |
