# Supplementary material for: CRISPR/Cas9 gene editing for the creation of an MGAT1-deficient CHO cell line to control HIV-1 vaccine glycosylation
Source: PLoS Biol. 2018 Aug 29;16(8):e2005817. doi: 10.1371/journal.pbio.2005817 (PMC6133382; doi:10.1371/journal.pbio.2005817)
Supplement: S1 Text — (DOCX) [file pbio.2005817.s005.docx]

IDEXX BioResearch Biological Material PCR Testing Capabilities Overview and Quality Assurance

Experience:

IDEXX BioResearch has over 40 years of experience in providing diagnostic services with expertise in molecular diagnostics polymerase chain reaction (PCR) services including biological materials testing, cell line authentication genetic testing, and microbiological services which form the cornerstone of our biologic materials testing. In addition to these services, serology, clinical pathology, and anatomic pathology services are provided for animal health monitoring. IDEXX BioResearch currently serves over 4000 clients worldwide including universities, research institutes, pharmaceutical companies, rodent producers, biotechnology companies, and the federal government.

**General Methodology:**

IDEXX BioResearch uses real-time PCR assays to detect an extensive list of viral, bacterial and fungal contamination of biological materials. The assay is designed and validated to have ≤ 10 copy sensitivity per PCR reaction to the agents in Table 1.

**Quality Assurance:**

IDEXX BioResearch uses strict quality control and quality assurance measures, in accordance with good laboratory practices, to ensure exceptional performance of all testing procedures. Quality control practices include: Experience, training, redundancy, and use of proper equipment and conditions.

1. **Experience** – The molecular diagnostic laboratory at IDEXX BioResearch has over a decade of experience in biological material testing, and more than 40 years of experience designing and validating assays to detect an extensive list of agents.
2. **Real-Time PCR Assay Validation:** All Real-Time PCR assays are put through an extensive validation process. Assays are designed utilizing all genome sequences deposited in GenBank or from sequence information generated in-house for organisms with limited or no genome sequences available from public databases. Assays are designed to be specific for the intended target by choosing genes and genome regions unique to the intended target. In addition, to ensure robust assay sensitivity, the most conserved genome regions of the intended target are chosen such that the primer and probe binding regions are areas less likely to be altered by potential genetic variation among field strains of the intended organism. Assays must pass analytical validation when tested in triplicate against dilutions of 1) a known positive control and 2) a known positive clinical case sample with the following criteria being met and reproduced on different run days: amplification efficiency of 95-105%, linearity over 5 points, calculated coefficient of variation (CV) of crossing points (Cp) equal to or smaller than 3%, CV calculated with absolute values equal to or smaller than 20%, r2 value equal to or larger than 0.993, signal to noise ratio of fluorescent signal: ≥10 and analytical sensitivity of 10 molecules or less per PCR reaction. Assays must pass clinical validation with the assay being tested against clinical samples characterized as positive or negative by another acceptable method. These methods will vary depending on diagnostic methodologies available for the organism of interest and include IDEXX BioResearch conventional PCR assays, sequence analysis, microbiologic methods (*i.e.,* Vitek, MALDI-TOF), microscopic examination or ATCC deposited isolates. Sequence analysis is performed on select positive samples to confirm amplification of the intended target. The assay passes the validation phase if it is proven to be sensitive and specific for the intended target(s).
3. **PCR reaction setup:** Two separate dedicated rooms are used for PCR reaction set up. The first is the “Clean Room” which is an isolated room maintained under positive air pressure where PCR reagents (enzymes mixes, buffers, primers and probes) are stored and has three dedicated hoods where PCR master mixes are added to reaction plates. Test sample nucleic acids and positive controls are never allowed into this room, preventing any exposure of these reagents to potential contaminating nucleic acids. The second room is the “Real-Time PCR Room” that has dedicated hoods for performing reverse transcription (RT) reactions to convert RNA into cDNA (hence forth referred to as DNA) and adding test sample DNA to PCR reaction plates. The “Real-Time PCR Room” also contains three networked real-time PCR machines that thermocycle 384 well PCR plates.
4. The PCR reaction master mixes and appropriate control reaction master mixes (PCR primers, TaqMan probe, Hot Start polymerase, buffers and water) are assembled in the “Clean Room” under sterile conditions in a dedicated hood. Like PCR assays and appropriate controls are grouped together on the test plate for efficient set up. Each real-time PCR reaction is performed individually in a 384 well plate. Once all PCR master mixes have been added, the plates are sealed and transported to the “Real-Time PCR Room”. Here, under a dedicated hood, test sample template DNA is added to the appropriate wells of the 384-well plate. In addition to each sample being tested for the agent(s) of interest, it is also tested by another PCR assay (extraction control) to make sure the sample contains amplifiable nucleic acids and does not contain PCR inhibitors. All handling of DNA is done in dedicated hoods under sterile conditions using filter barrier aerosol resistant tips. Hoods and work surfaces are decontaminated after each use. For RNA agents, RNA is reverse transcribed to cDNA under sterile conditions in a dedicated hood in the “Real-Time PCR Room” prior to addition to the real-time PCR reaction plate. In addition to the individual sample extraction control mentioned above, positive and negative control reactions are performed for each test assay to ensure assay performance. After the addition of all template DNA, the PCR plates are sealed, centrifuged and placed in a thermocycler.
5. **Thermocycling and target detection:** The laboratory is equipped with four networked real-time thermocyclers that can perform 384 PCR reactions per hour. Data from all PCR runs are saved on the thermocyclers and on a secure networked server that is backed up daily. After rapid thermocycling, the data from the PCR reactions are interpreted. If the test sample contains DNA for the specific pathogen of interest, the PCR primers will bind and generate a PCR amplicon the length (in nucleotide bases) of the distance between the two primers. In addition, during the generation of the specific PCR product, the real-time PCR probe (which is also specific for the agent of interest) is hydrolyzed, releasing a fluorescent signal that is measured after each cycle of the PCR reaction. Positive reactions generate a sigmoidal fluorescent curve and negative reactions produce no curve; collectively these signals are referred to as amplification plots. The PCR cycle number at which a positive signal rises above baseline is called the crossing point or Cp value. Samples with larger amounts of target DNA present produce a florescent signal after fewer PCR cycles (*i.e.*, have a lower Cp value) than samples that contain lower amounts of target DNA (*i.e.*, have a higher Cp value).
6. **PCR data interpretation:** The thermocycler software makes a determination on each reaction as being positive or negative, but occasionally it can make the wrong determination. Thus, the amplification plot of each test and control reaction is reviewed by two separate individuals: 1) the technician performing the PCR does the primary review of the data and 2) a select group of senior PCR technicians, the PCR laboratory supervisor or the PCR laboratory director performs the secondary review of the data. For the given test being run, the positive control must be positive and the negative control must be negative. If these criteria are not met the test is considered a “no test” and the testing is repeated. For each test sample, the extraction control must show that there is amplifiable DNA in the test sample. If this criteria in not met, DNA or RNA is re-extracted from remaining original material and the PCR testing is repeated. If all of the quality control criteria are met, then the individual sample reactions are evaluated for positive or negative status. Any sample generating a positive signal is noted and retested to confirm the positive result. In the retesting of positive samples, if the secondary testing confirms the positive result then the sample is turned out as positive. If the secondary testing does not confirm the original result, then the extracted nucleic acid is subjected to additional repeat testing or nucleic acid is re- extracted from remaining original sample material and repeat testing is performed until a clear disposition of the sample as positive or negative can be made. Any sample producing no signal is interpreted as a negative test result.
7. **PCR Quality Control and Assurance:** The exquisite sensitivity of PCR requires extensive quality control measures to prevent cross-contamination between samples. A number of general procedures are in place to address this issue. First, IDEXX BioResearch has dedicated laboratories for each of the following PCR activities: sample preparation, nucleic acid extraction, PCR mastermix and plate setup and template addition. There is also a dedicated area for post-PCR analysis when conventional PCR’s are run or sequence analysis is performed and a dedicated room under negative air pressure for maintaining concentrated positive controls. Moreover, the room where PCR mastermix and plate setup occurs houses all primers, probes and reagents for the PCR reactions and is under positive pressure; sample template never enters this room. Each of these laboratories has dedicated equipment that remains in these areas including technician lab coats. To avoid contamination of PCR reactions with previously amplified nucleic acids, dUTP and uracil N-glycosylase (UNG) are used in real-time PCR mastermixes rendering previously DNA amplified non-amplifiable. Technicians wear disposable gloves that are changed frequently and are disposed of and replaced with new gloves when moving between the different laboratory areas. All samples are handled under sterile conditions throughout sample preparation, nucleic acid purification and PCR setup. This includes the use of sterile reagents, sample tubes, sample prep trays, scalpel blades and filter-barrier pipette tips. In addition, when samples are being prepared before nucleic acid extraction, only one sample is prepared at a time. Sample holding trays are decontaminated by UV irradiation after each use and individual sterile disposable pads are placed over the work surface while samples are prepared before nucleic acid extraction. All hoods are decontaminated by UV irradiation and bleach after each use and other laboratory work surfaces are decontaminated with bleach after each use. The use of robotics during nucleic acid extraction also reduces the chance of cross- contamination. To reduce any chance of environmental contamination contributing to a false positive PCR result, the real-time PCR laboratory is monitored weekly for environmental contamination of amplifiable nucleic acid for each of the assays run in the laboratory. Areas that are found to contain amplifiable DNA are decontaminated and then retested until the area tests negative.
8. **PCR Controls:** Multiple controls are used when performing the PCR reactions. For each sample tested, in addition to the requested assays, an additional PCR is set up to ensure that the test sample contains amplifiable DNA or cDNA for the

respective sample type. This ensures that nucleic acid extraction was successful, there is no inhibition of the PCR reaction, the reverse transcriptase step (if performed) was successful and PCR setup was performed correctly. Each run of a specific PCR assay includes a positive control with a low number of template copies providing a robust control of assay performance and a reaction that contains no template DNA or cDNA to ensure the PCR reagents are not contaminated with any template nucleic acids. Each lot of PCR reagents is tested for performance against low copy number positive controls to ensure that new reagent lots maintain established assay sensitivity. This is accomplished by measuring the crossing point (Cp) values of each of the real-time PCR assays. The real-time thermocyclers give error codes if the thermocycling parameters on any run fall outside of factory set specifications. All pipettes used in the laboratory are routinely calibrated. Freezers used to store PCR reagents, samples and extracted nucleic acid are equipped with temperature probes that are connected to the Rees Centron Environmental Monitoring System. This system is designed to meet AABB, AAALAC, FDA, GxP, GAMP, USDA and USP regulatory requirements by constantly monitoring critical equipment, and provides centralized data collection, automatic reporting and around-the-clock alarm notification should the environment fall outside of compliance.

9. **Confirmation of PCR results:** Because of the exquisitely high sensitivity of PCR assays, there is the potential for cross- contamination of samples and the generation of a false positive test result. The laboratory has developed a comprehensive set of practices described above to minimize the potential for false positive test results. To further reduce false positive test results, all positive PCR tests are re-tested by performing a second PCR with the same or alternate PCR primers and probe. False negative results can occur for several reasons. The most common reason for false negative reactions is failure to eliminate substances that are inhibitory to the PCR reaction from the purified nucleic acids. After extensive efforts and validation, IDEXX BioResearch has developed protocols for isolation of nucleic acids from an array of sample types, including tissues and feces that yield sensitive PCR and RT-PCR reactions and allow detection of microorganisms present at very low abundance (≤10 organisms). False negative reactions can also occur due to improper sample or reagent storage during shipping or while in the laboratory. The extraction controls mentioned above are used to ensure amplifiable nucleic acids that lack inhibitory substances are present in the test samples. All samples received are checked for integrity (*i.e.,* labeling, container damage, leakage, arriving thawed, *etc*.). Our quality checks on reagents minimize the chance of obtaining faulty reagents from the manufacturer; sample and reagent storage temperatures are monitored in real-time using the Rees Centron Environmental Monitoring System as described above and sample handling procedures in the laboratory are designed to preserve samples and reagents. The use of low copy positive controls with every run of a specific PCR assay provides a sensitive indicator of proper assay performance. The real-time PCR gives an objective output [crossing point (Cp) value] of these positive controls allowing for sensitive day-to-day monitoring of assay performance.

10. **Additional Procedures relevant to Real-Time Testing**

a. **Nucleic acid purification:** The laboratory has protocols to process and extract DNA and RNA from a variety of samples including, but not limited to solid tissues, feces, blood, serum, ascites, swabs (of the animal or environment), Matrigel, cultured cells, blood, formalin-fixed paraffin embedded tissue and cultured bacteria. Two nucleic acid extraction robots that utilize a silica-coated magnetic bead technology are used for most DNA and RNA purifications. These dedicated robots are specifically able to efficiently isolate high quality, inhibitor-free nucleic acids while maintaining sample integrity and preventing cross-contamination of samples. If needed, the laboratory is also equipped to purify DNA and RNA manually using spin column technology which we routinely use when extracting nucleic acids from formalin fixed paraffin embedded tissue.

b. **PCR data reporting:** After each test is completed and has passed review by two separate individuals, the PCR technicians record the testing results in the case file. In addition, the bar code specific test data are electronically uploaded into the IDEXX BioResearch LIMS and become associated with the correct case/test sample. When all of the required tests for the case are completed and reviewed, a preliminary report is generated. This case file along with the newly generated report and the original PCR data are critically reviewed by a senior technician, the IDEXX BioResearch PCR laboratory supervisor, Dr. Mariana Morales-Quinones, or Dr. Robert Livingston to ensure that the data has been interpreted correctly and that all of the data from the original submitted paperwork is accurately reflected in the report. Approved case results are made immediately available to the designated institutional personnel.

c. **PCR sample retention and storage:** All extracted nucleic acids and unused original sample material are frozen and are retained for at least six months or one month, respectively. These samples are available for additional testing.

d. **PCR Deviation Investigation Process:** Given that positive and negative controls are run with all PCR assays, it is clear when the tests do not perform as expected. Depending on the nature of the test failure different courses of action are taken:

i. Should all PCR assays perform outside of normal testing parameters then reagents and equipment common to all tests are investigated as the root cause (e.g. extraction robots and chemistries, thermocyclers and PCR enzyme master-mix reagents). Given that we have redundancies in the lab including at least two of every machine (thermocyclers and extraction robots), machine malfunctions are easily identified. In addition, we maintain working stocks and back-up stocks of all reagents so reagent problems can be easily investigated and identified. ii. As the PCR reagents are checked for quality upon receipt and nearly all tests, including appropriate controls with each run, are performed on a daily basis, testing deviations are rare. However, when individual assay controls do not perform as expected, the first course of action is to repeat the PCR test which usually resolves the issue. In instances when this does not fix the problem, fresh back-up aliquots of primers and probes are used to run the assays which nearly always resolve the issue. In the course of supervising the PCR laboratory for over 13 years, we have always been able to identify and expediently resolve test deviation issues.
